# Influenza breakthrough infection in vaccinated mice is characterized by non-pathological lung eosinophilia

**DOI:** 10.3389/fimmu.2023.1217181

**Published:** 2023-08-04

**Authors:** Lauren A. Chang, Angela Choi, Raveen Rathnasinghe, Prajakta Warang, Moataz Noureddine, Sonia Jangra, Yong Chen, Bruno G. De Geest, Michael Schotsaert

**Affiliations:** ^1^ Department of Microbiology, Icahn School of Medicine at Mount Sinai, New York, NY, United States; ^2^ Graduate School of Biomedical Sciences, Icahn School of Medicine at Mount Sinai, New York, NY, United States; ^3^ Global Health and Emerging Pathogens Institute, Icahn School of Medicine at Mount Sinai, New York, NY, United States; ^4^ Department of Pharmaceutics, Ghent University, Ghent, Belgium

**Keywords:** eosinophils - immunology, influenza, breakthrough infection, eosinophils subtypes, influenza vaccination

## Abstract

Eosinophils are important mediators of mucosal tissue homeostasis, anti-helminth responses, and allergy. Lung eosinophilia has previously been linked to aberrant Type 2-skewed T cell responses to respiratory viral infection and may also be a consequence of vaccine-associated enhanced respiratory disease (VAERD), particularly in the case of respiratory syncytial virus (RSV) and the formalin-inactivated RSV vaccine. We previously reported a dose-dependent recruitment of eosinophils to the lungs of mice vaccinated with alum-adjuvanted trivalent inactivated influenza vaccine (TIV) following a sublethal, vaccine-matched H1N1 (A/New Caledonia/20/1999; NC99) influenza challenge. Given the differential role of eosinophil subset on immune function, we conducted the investigations herein to phenotype the lung eosinophils observed in our model of influenza breakthrough infection. Here, we demonstrate that eosinophil influx into the lungs of vaccinated mice is adjuvant- and sex-independent, and only present after vaccine-matched sublethal influenza challenge but not in mock-challenged mice. Furthermore, vaccinated and challenged mice had a compositional shift towards more inflammatory eosinophils (iEos) compared to resident eosinophils (rEos), resembling the shift observed in ovalbumin (OVA)-sensitized allergic control mice, however without any evidence of enhanced morbidity or aberrant inflammation in lung cytokine/chemokine signatures. Furthermore, we saw a lung eosinophil influx in the context of a vaccine-mismatched challenge. Additional layers of heterogeneity in the eosinophil compartment were observed via unsupervised clustering analysis of flow cytometry data. Our collective findings are a starting point for more in-depth phenotypic and functional characterization of lung eosinophil subsets in the context of vaccine- and infection-induced immunity.

## Introduction

Influenza is an Orthomyxovirus composed of 8 single-stranded negative-sense RNA segments which encode 11 proteins, one of which is an error-prone polymerase that is conducive to gradual genetic mutations and subsequent drift in the major influenza antigenic determinants: hemagglutinin (HA) and neuraminidase (NA) (1–4). Influenza is responsible for seasonal epidemics as well as global pandemics, such as the 1918-1919 pandemic and 2009 pandemic (1, 2). The World Health Organization estimates the annual global disease burden of influenza to be 1 billion infections, resulting in approximately 300,000 to 500,000 deaths and many more hospitalizations every year (5).

Vaccination is a key strategy in mitigating influenza transmission and disease severity (1, 6, 7). Currently clinically approved vaccines include live attenuated and inactivated split vaccines, administered with or without adjuvant in a wide variety of doses depending on the target demographic (1). Inactivated influenza vaccines (IIV) are the most commonly administered type of seasonal influenza vaccine and are comprised of three or four vaccine viruses: one influenza A H1N1 virus, one influenza A H3N2 virus, and one or two influenza B viruses from either or both clades (3). IIV viruses are propagated in embryonated chicken eggs or in cell culture with Madin-Darby Canine Kidney (MDCK) cells, followed by inactivation and purification, then split using a detergent (3). Vaccination with seasonal IIV confers sufficient protection against severe disease and mortality, however efficacy is dependent on concordance between candidate vaccine strains and circulating strains alongside host-intrinsic factors such as sex, age, and the presence of comorbidities which may skew the nature of the inherent immune response to vaccination and infection (4, 8–15). The low to medium efficacy of IIV can also be partially attributed to the nature of the immune responses generated by inactivated viral vaccines (8, 16). IIV elicits the generation of binding and neutralizing antibodies, predominantly to the surface glycoproteins HA and NA, but often is a poor inducer of antigen-specific T cells, which are critical effector cells during influenza infection when the neutralizing antibody response is insufficient in controlling replication (17–20). IIV is also administered intramuscularly, resulting in little to no generation of first-line mucosal immunity, such as secretory IgA, in the upper and lower respiratory tract required for robust protection against respiratory viral infection (21–26). As a result, breakthrough infection with circulating influenza strains is common in vaccinated individuals (27). T cells can mitigate the development of severe disease in the event of breakthrough infection through recognition and killing of infected cells presenting peptides from supra-seasonally conserved viral antigens, such as nucleoprotein (NP), through cytotoxic activity (21, 28, 29). The contribution of myeloid cells to host immunity after breakthrough infection in vaccinated hosts is understudied, especially in the context of granulocytes like eosinophils.

Eosinophils are crucial cellular players in Type 2 host immunity at mucosal surfaces, most prominently known for their role in anti-helminth responses, allergy, and asthma (30–37). Additional studies have shed light on the additional roles for eosinophils outside of Type 2 immunity during viral, fungal, and bacterial infections, both as mediators of protection or immunopathology *in vivo* (38–45). Lung eosinophils are able to participate in effective antiviral responses against respiratory syncytial virus (RSV), parainfluenza virus, and influenza, both as direct mediators of antiviral activity or as support to other cells via secreted factors or surface ligands (38–43). In the context of viral infection in vaccinated hosts, eosinophils have been most notably linked to the immunopathology of vaccine-associated enhanced respiratory disease (VAERD), as observed in the formalin-inactivated RSV (FI-RSV) vaccine trials in infants during the 1960s (46–52). Other preclinical models for severe acute respiratory syndrome coronavirus (SARS-CoV), SARS-CoV-2, and influenza across different vaccine modalities have demonstrated eosinophilic immunopathology or VAERD (53–57). In contrast, an ultraviolet (UV)-inactivated whole-virion SARS-CoV vaccine in BALB/c mice also induced eosinophil infiltration into the lungs upon viral challenge, although morbidity and clinical illness presentation were comparable to a TLR7/8 agonist adjuvanted version of the vaccine, which did not induce eosinophilia (58). While studies investigating FI-RSV have identified eosinophil infiltration as a hallmark of VAERD, with a notable contribution from CD4 T cells and Th2 cytokines to the overall phenotype, no mechanistic understanding of how vaccine-induced eosinophil recruitment directly mediates protection from or enhance disease exists yet (51, 59, 60).

A growing body of literature has described an emerging role for eosinophils in protection and lung recovery following viral infection (31, 37, 39, 61). Further phenotypic heterogeneity within eosinophils and plasticity among other granulocytes has also been elucidated (62–71). For example, Mesnil et al. have described two major subsets of eosinophils in the mouse lung, an inflammatory subset (iEos) recruited in an IL-5-dependent manner which are CD101^+^ CD62L,^-^ and a tissue-resident subset (rEos) which are CD101^-^ CD62L^+^ and possesses regulatory capabilities (62, 72). Lung eosinophils can also be bifurcated on the basis of Siglec-F expression, with Siglec-F^hi^ eosinophils corresponding to iEos and Siglec-F^int^ eosinophils corresponding to rEos (62). Functionally, iEos have been associated with exacerbation of Type 2 responses while rEos play a more role and can attenuate aberrant Th2 responses in both mouse models and human samples for asthma (37, 62, 66, 68, 73, 74). How these individual subsets are implicated outside of Type 2 immunity during respiratory viral infection and vaccination is not well defined. Furthermore, it is not yet known which subsets are implicated in vaccine-associated lung eosinophilia, if at all, and downstream protection or pathogenesis during respiratory viral infection.

In a vaccine-matched sublethal (0.2 LD_50_; 0.2X the lethal dose required for mortality of 50% of animals in the group) influenza infection mouse model designed to mimic breakthrough infections in immune-experienced hosts, we previously reported a dose-dependent recruitment of eosinophils upon virus challenge, with triple-vaccinated mice exhibiting greater absolute numbers of eosinophils in the lungs than single-vaccinated mice at 7 days post-challenge (DPC) (75). Interestingly, lung eosinophilia was not associated with enhanced disease or morbidity but with protection (75, 76). Here, we conducted follow-up studies to investigate the phenotype of the lung eosinophils, any potential effects of adjuvant, and the impact of pre-existing heterosubtypic immunity from viral challenge on eosinophil recruitment, providing new insights on the role of eosinophils and its subsets in vaccine-mediated protection during breakthrough influenza infection.

## Methods

### Study design

Female, 6-8 week old BALB/c mice obtained from The Jackson Laboratory (Bar Harbor, ME) and housed under specific pathogen-free conditions with food and water provided *ad libitum*. All experiments described herein were approved by and performed according to the Icahn School of Medicine at Mount Sinai Institutional Animal Care and Use Committee. The following reagent was obtained through BEI Resources, NIAID, NIH: Fluzone® Influenza Virus Vaccine, 2005-2006 Formula, NR-10480. Mice were vaccinated intramuscularly in the hind legs with a seasonal trivalent inactivated influenza virus vaccine (TIV; Fluzone® Influenza Virus Vaccine) containing an influenza A H1N1 component (A/New Caledonia/20/1999/IVR-116), influenza A H3N2 component (A/New York/55/2004/X-157 [an A/California/7/2004-like strain]), and influenza B component (B/Jiangsu/10/2003 [a B/Shanghai/361/2002-like strain]).

For the first study assessing the impact of adjuvant on lung immune cell dynamics, TIV was adjuvanted with one of the following: no adjuvant, 2% alhydrogel (alum, Invivogen), or IMDQ-PEG-CHOL (abbreviated to IMDQ in this paper). IMDQ is a lymph node-targeting amphiphilic conjugate of an imidazoquinoline TLR7/8 agonist linked to poly(ethylene glycol) and cholesterol (77–79). A control group receiving alum adjuvant alone was included for comparison to the other TIV groups. An OVA-sensitized positive control group for lung eosinophilia and a PBS control were included in the study as well. 5 mice were used per treatment group.

For the second study assessing the impact of vaccine-mismatched heterosubtypic sublethal challenge on lung myeloid cell dynamics, TIV was adjuvanted with alum. A group receiving alum alone was included as a negative control. 3-4 mice were used per treatment group.

TIV and adjuvant mixtures were administered intramuscularly to both hind legs (50 µL/leg, 100 µL/mouse total).

### Influenza challenge

Mice were anesthetized using an intraperitoneal (i.p.) injection of a ketamine and xylazine mixture, then challenged intranasally (i.n.) with 50 µL of mouse-adapted egg-grown influenza viruses or egg allantoic fluid as vehicle control. Body weights were monitored for 7-10 days post-infection to assess morbidity. 100% body weight was defined as the weight of the mouse immediately before the challenge. The viruses used were H1N1 A/New Caledonia/20/1999 (NC99) and H3N2 A/X-31 at a sublethal (0.2 LD_50_) challenge dose.

### OVA sensitization

Mice received a 100 µL i.p. injection containing 20 µg of Imject™ ovalbumin (OVA, Thermo Scientific) adsorbed to alum on days 0 and 7. On day 14, mice were anesthetized as described above and then challenged i.n. with 20 µg of Imject™ ovalbumin in PBS, diluted to a final volume of 50 µL/mouse.

### Serum collection

Blood was collected via the submandibular route and then allowed to coagulate at 4°C overnight. Coagulated blood was then spun down at 400 x *g* for 5 minutes at 4°C and serum was collected and stored at -20°C until further analyses were performed.

### Flow cytometry

Lung lobes were collected in 3 mL of RPMI-1640 media supplemented with 10% heat-inactivated fetal bovine serum (FBS) and 1X Penicillin-Streptomycin. Lung tissue was minced in 6-well plates using surgical scissors and then digested with Collagenase D at a working concentration of 2 mg/mL for 15 minutes at 37°C while shaking. After digestion, single-cell suspensions were generated by forcing digested tissue through a 70 µm cell strainer with the flat end of a 1 mL syringe plunger in a new 6-well plate. Wells were washed with an additional 3 mL of media then transferred to a 15 mL conical vial and centrifuged at 400 x *g* for 5 minutes at 4°C. Supernatants were discarded then pellets were resuspended in 5 mL of 1X ammonium chloride red blood cell lysis buffer and incubated at room temperature for 5 minutes. Cells were centrifuged and pellets were resuspended in 5 mL of 1X PBS to wash. After centrifugation, cell pellets were resuspended in 50 µL of Purified Rat Anti-Mouse CD16/CD32 Fc Block (clone 2.4G2, BD) diluted 1:100 in Staining Buffer (1% bovine serum albumin and 2 mM EDTA in 1X PBS), transferred to a 96-well V-bottom plate, and incubated at room temperature for 5 minutes. After incubation, 50 µL of surface stain antibody cocktail comprised of the following antibodies and dyes were added to cells: CD11c FITC (1:150, clone HL3, BD), CD125 PE (1:150, clone T21, BD), Siglec-F PE-CF594 (1:150, clone E50-2440, BD), Ly6G PerCP-Cy5.5 (1:150, clone 1A8, BD), CD101 PE-Cy7 (1:150, clone Moushi101, Invitrogen), CD11b APC (1:150, clone M1/70, BioLegend), MHC II Alexa Fluor 700 (1:150, clone M5/114.15.2, Invitrogen), CD62L APC-Cy7 (1:150, clone MEL-14, BD), Fixable Viability Dye eFluor 450 (1:200, eBioscience) for the eosinophil phenotyping study; or CD11b APC (1:100, clone M1/70, BioLegend), CD11c FITC (1:100, clone HL3, BD), MHC II eFluor™ 450 (1:200, clone M5/114.15.2, Invitrogen), Siglec-F (1:100, clone E50-2440, BD), CD64 PE (1:100, clone X54-5/7.1, BD), BD Horizon™ Fixable Viability Stain 700 (1:200, BD) for the vaccine-mismatched challenge study. After incubating in the dark for 20 minutes at room temperature, cells were washed by adding 120 µL of Staining Buffer on top of the cells, followed by centrifugation, discarding supernatants, resuspension of pellets in 200 µL Staining Buffer, and centrifugation. After discarding supernatants from the last wash, pellets were resuspended in 200 µL of Staining Buffer and 5 µL of CountBright Absolute Counting Beads (ThermoFisher) were added to all samples, excluding single-stained controls and the unstained lung cell control, to facilitate quantification of absolute cell numbers. UltraComp eBeads Plus Compensation Beads (ThermoFisher) were used to create single-stained compensation controls. Samples were acquired using a Beckman Coulter Gallios flow cytometer with Kaluza software. Data analysis was performed using FlowJo 10.8.1 (Treestar) and compensated using the built-in AutoSpill algorithm. Data were visualized using Graphpad Prism version 9.4.1. Unsupervised clustering analyses were conducted in the R computing language (ver. 4.05) using the *CATALYST* (Cytometry dATa anALYSis Tools) package version 1.20.1 in RStudio (ver. 1.4.1106) (80).

### Determination of lung viral titers

Lung left lobes were collected in 500 µL of 1X PBS in prefilled homogenizer bead tubes containing 3.0 mm high impact zirconium beads (Benchmark Scientific), snap-frozen on dry ice on the day of harvest, then stored at -80°C. On the day of the assay, samples were homogenized and then centrifuged at 10,000 x *g* for 5 minutes at 4°C after thawing. Clarified supernatants were serially diluted 10-fold, beginning at 1:10 for a total of 6 dilutions in 1X PBS. In a 12-well tissue culture plate seeded the day prior with Madin-Darby Canine Kidney (MDCK) cells, wells were washed twice with 1 mL of 1X PBS per well. After washing, 150 µL of each clarified lung supernatant dilution was added to one well and then incubated for 1 hr at room temperature, rocking plates every 10 minutes. After incubation, cells were washed once with 1 mL/well of 1X PBS then 1 mL/well of overlay (2% Oxoid agar in sodium bicarbonate buffered serum-free 2X minimum essential medium (MEM) supplemented with 1% diethylaminoethyl (DEAE)-dextran and 1 μg/mL tosylamide-2-phenylethyl chloromethyl ketone (TPCK)-treated trypsin) was added to each well. After allowing the overlay to solidify at room temperature for 15 minutes, plates were incubated at 37°C for 48 h and fixed with 1 mL of 4% formalin per well overnight at 4°C. After removal of the fixative and agar overlay, plates were washed with PBS-T (PBS with 0.05% Tween 20). Monolayers were immunostained with 150 μL/well hyperimmune rabbit polyclonal serum diluted in 5% milk in PBS-T overnight at 4°C on a plate rocker. Plates were washed 3 times with PBS-T then 150 μL/well of horseradish peroxidase (HRP)-conjugated anti-rabbit IgG Fc secondary antibody diluted in 5% milk in PBS-T for 1 hr at room temperature while rocking. Plates were washed, then 150 μL/well KPL TrueBlue was added and incubated at room temperature for 30 minutes on a plate rocker. Plates were washed and plaques were counted to determine lung viral titers.

### Measurement of cytokines and chemokines

Lung left lobes were collected, homogenized, and clarified as described above. We used the Cytokine & Chemokine 26-Plex Mouse ProcartaPlex™ Panel 1 (ThermoFisher) to measure cytokine and chemokine concentrations in the lungs. The assay was conducted according to the manufacturer’s instructions and the plate was placed on an orbital shaker set to 300 rpm for all incubation steps. Briefly, 25 µL of lung homogenate was mixed with 25 µL of Universal Assay Buffer provided by the kit, then combined with beads in an optical bottom black 96-well plate and incubated for 30 minutes at room temperature protected from light. After 30 minutes, the plate was moved to 4°C for overnight incubation. The next day, the plate was equilibrated to room temperature for 30 minutes, then washed 3 times with 150 µL/well of 1X Wash Buffer diluted according to kit instructions. Following washing, 25 µL/well of 1X Detection Antibody mixture was added and incubated at room temperature for 30 minutes. The plate was washed 3 times then 50 µL/well of 1X Streptavidin-PE solution was added and incubated for 30 minutes at room temperature. After washing the plate 3 times, 120 µL/well of Reading Buffer was added and the plate was incubated for 5 minutes at room temperature. Subsequently, data were acquired on a Luminex 100/200 analyzer (Millipore) with xPONENT software (version 4.3). Data visualization and analysis were conducted using GraphPad Prism (version 9.4.1) and R computing language (ver. 4.05) in RStudio (ver. 1.4.1106).

### Enzyme linked immunosorbent assays

To measure vaccine-specific total IgG, Nunc MaxiSorp 96-well plates (ThermoFisher) were coated with 100 µL/well TIV diluted 1:100 in carbonate-bicarbonate buffer and incubated overnight at 4°C. Plates were washed 3 times with PBS-T then blocked with 200 µL/well of blocking buffer (5% milk in PBS-T) and incubated for 1 h at room temperature. During the blocking step, sera were serially diluted 4-fold a total of 7 times starting from a 1:100 dilution for total IgG. After blocking, plates were washed 3 times then 100 µL/well of diluted sera was added to plates and incubated for 1 h at room temperature. Plates were washed then 100 µL/well of goat anti-mouse IgG-HRP (Cat. No. ab6823, Abcam) diluted 1:5000 in blocking buffer was added to each well and incubated for 1 h at room temperature. After washing plates 3 times, 100 µL/well of 1-Step Turbo TMB Substrate (ThermoFisher) was added and then incubated for 20 minutes at room temperature. The reaction was stopped by adding 100 µL/well of ELISA Stop Solution (Invitrogen) and plates were read at 450 nm and 650 nm. Background subtracted optical density values (OD_450-650nm_) were used for downstream analyses.

To measure total IgE, Nunc MaxiSorp 96-well plates (ThermoFisher) were coated with 100 µL/well of anti-mouse IgE antibody (Clone R35-72, BD) diluted to a concentration of 2 µg/mL in carbonate-bicarbonate buffer and incubated overnight at 4°C. Similar to the IgG ELISA described above, plates were washed the next day 3 times with PBS-T and then blocked with 200 µL/well of blocking buffer for 1 h at room temperature. As plates were incubated with blocking buffer, sera were diluted 4-fold a total of 7 times, starting from a 1:50 dilution. Unlabelled purified mouse IgE (SouthernBiotech) diluted 4-fold a total of 11 times from a starting concentration of 400 ng/mL to generate a standard curve. Plates were washed after blocking then 50 µL/well of diluted sera was added to plates and incubated for 1 h at room temperature. After incubation with sera, plates were washed and then incubated with 100 µL/well of goat anti-mouse IgE-HRP (SouthernBiotech) for 1 h at room temperature. Plates were then washed, developed, and read as described for the IgG ELISA above after incubation. Subtracted optical density (OD) values of the 1:50 dilution were used to determine serum concentrations of total IgE.

All data were analyzed using GraphPad Prism (version 9.4.1).

### Hemagglutination inhibition

6 µL of serum was pretreated with 18 µL of receptor destroying enzyme II (Seiken) and incubated at 37°C overnight. After incubation, 18 µL of 2.5% sodium citrate solution was added and incubated at 56°C for 30 minutes followed by an addition of 18 µL of 1X PBS. Pretreated sera were stored at 4°C until use in the assay. Hemagglutination (HA) units were calculated via HA assay by combining a serial 2-fold dilution of stock virus in PBS with an equal volume of 0.5% turkey red blood cells (RBC) and incubated at 4C for 45 minutes. For the HA inhibition (HAI) assay, 50 µL of pretreated sera were serially diluted 2-fold a total of 11 times in PBS. Stock virus was diluted to a concentration of 8 HA units per 50 µL, then 25 µL/well of diluted virus was added to all serum-containing wells. Plates were incubated at room temperature for 30 minutes, then 50 µL/well of 0.5% turkey RBC was added to all wells. After incubation at 4°C for 45 minutes, HAI titers were recorded.

### Principal component analysis

The following metrics from each individual mouse were integrated into one data frame for principal component analysis (PCA): flow cytometry absolute numbers for 18 cell populations of interest; body weight percentages from 7 timepoints; 7 DPC lung viral titers; IgG and HAI titers from 14 days post-vaccination and post-challenge sera; total IgE concentrations post-vaccination and post-challenge sera; concentrations of 26 cytokines/chemokines in clarified lung homogenate supernatants. Data visualization and analysis were conducted using *prcomp* in the R computing language (ver. 4.05) in RStudio (ver. 1.4.1106).

## Results

### Pulmonary eosinophilia is observed upon virus challenge only in vaccinated mice during breakthrough influenza infection, irrespective of adjuvant, and the composition of pulmonary infiltrates is phenotypically similar to those in OVA-sensitized mice

**Figure 1:**
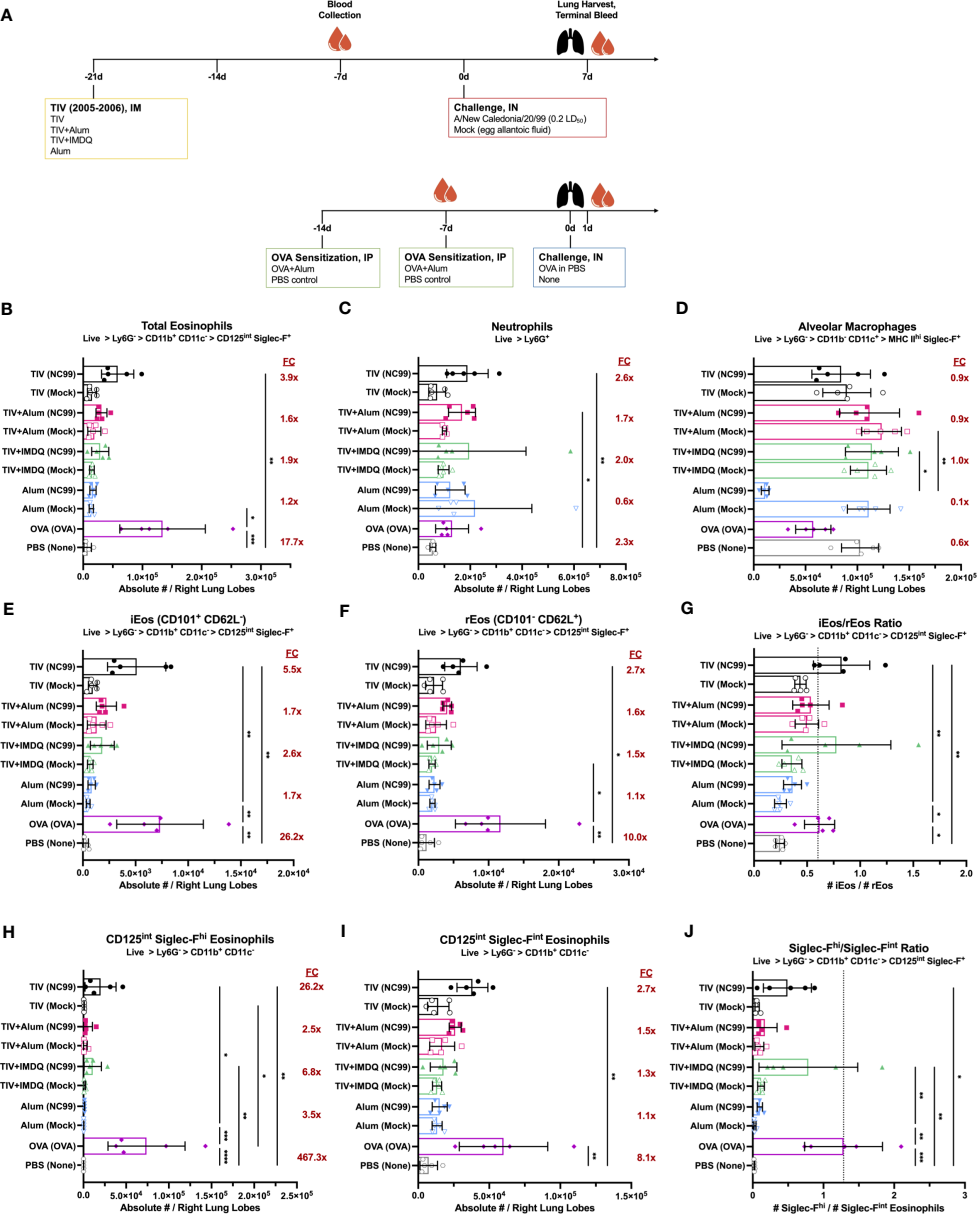
Lung eosinophil, neutrophil, and alveolar macrophage dynamics are modulated by influenza vaccination and challenge, irrespective of adjuvant. **(A)** Outline of study assessing impact of adjuvant on immune cell dynamics after vaccination and challenge. Absolute number of **(B)** total eosinophils, **(C)** neutrophils, **(D)** alveolar macrophages, **(E)** inflammatory eosinophils (iEos, CD101^+^ CD62L^-^), and **(F)** resident eosinophils (rEos, CD101^-^ CD62L^+^) in right lung lobes. Ratio of **(G)** iEos/rEos. Absolute number of **(H)** Siglec-F^hi^ and **(I)** Siglec-F^int^ eosinophils in right lung lobes. Ratio of **(J)** Siglec-F^hi^/Siglec-F^int^ eosinophils. Ratios were calculated using the absolute numbers of each population in the right lung lobes. Red numbers in **(B–D, E, F, H, I)** indicate fold-change (FC) of NC99-challenged animals compared to mock-challenged animals within each vaccination condition. For **(B–J)**, bars indicate mean ± standard deviation (SD). Statistical significance in **(B–J)** was determined via Kruskal-Wallis one-way ANOVA with Dunn’s multiple comparisons test: ****P < 0.0001, ***P = 0.0001 to 0.001, **P = 0.001 to 0.01, *P = 0.01 to 0.05.

We used the 2005-2006 Fluzone trivalent inactivated influenza vaccine (TIV) as our model vaccine. Given that the previous study by Choi et al. was conducted using TIV adjuvanted with alum (TIV+alum), a known Th2-skewing adjuvant, we included the following groups in the study to disentangle any potential adjuvant effects: TIV with no adjuvant, TIV adjuvanted with alum to match the original study (TIV+alum), TIV adjuvanted with a lymph node targeting amphiphilic conjugate of the imidazoquinoline TLR7/8 agonist connected to poly(ethylene glycol) and cholesterol to potently elicit Th1-skewed responses (TIV+IMDQ), and alum alone with no TIV to assess if any effects previously observed were inherently due to alum itself rather than the vaccine (**Figure 1A**) (75, 77, 78). Ovalbumin (OVA)-sensitized allergic mice were included positive control for lung eosinophilia, alongside a PBS negative control (**Figure 1A**). Three weeks following intramuscular (i.m.) vaccination with the described regiments, mice were challenged with a sublethal (0.2 LD_50_), vaccine-matched challenge with A/New Caledonia/20/1999 (NC99) or mock-challenged with egg allantoic fluid since all viruses in the study were propagated in embryonated chicken eggs. At 7 days post-challenge (DPC) for the vaccination groups and 1 DPC for the control groups, we quantified key lung immune cell populations of interest: eosinophils, neutrophils, and alveolar macrophages (**Figure S1**).

#### Total eosinophils

We observed an enrichment in the absolute number of total eosinophils (Ly6G^-^ CD11b^+^ CD11c^-^ CD125^int^ Siglec-F^+^) in all groups that were both TIV-vaccinated and NC99-challenged, but not in groups that were TIV-vaccinated and mock-challenged (**Figure 1B**). There was a 3.9-fold enrichment for eosinophils in the NC99-challenged, unadjuvanted TIV mice compared to the corresponding mock-challenged group. NC99-challenged, TIV+alum mice and TIV+IMDQ mice had a 1.6-fold or 1.9-fold enrichment of eosinophils, respectively, over their mock-challenged counterparts. In contrast, NC99-challenged alum-vaccinated mice had a 1.2-fold enrichment in eosinophils compared to the mock-challenged alum-vaccinated group, and comparable numbers of eosinophils as the unchallenged PBS control group. OVA-sensitized mice had a tremendous influx of eosinophils in the lung, characteristic of the model, with a 17.7-fold higher absolute number of eosinophils in the right lung lobes compared to the PBS control group, which received no intranasal challenge. There appeared to be no impact of adjuvant on the lung eosinophil numbers, as we observed enrichment for eosinophils, typically associated with Type 2 immunity, even in groups with no adjuvant (TIV) or with a strong Type 1-skewing adjuvant (TIV+IMDQ).

#### Neutrophils

Next, we examined lung neutrophil (Ly6G^+^) numbers (**Figure 1C**). Within the TIV-vaccinated mice, we observed a roughly 1.7 to 2.6-fold increase in neutrophils only in groups receiving NC99 challenge but not in mock-challenged mice. There was no enrichment of neutrophils observed in the mice that received alum-only as the priming vaccination dose, even when challenged with NC99. OVA-sensitized mice had 2.3-fold more absolute numbers of neutrophils compared to the unchallenged PBS control mice, similar to what we observed in the TIV-vaccinated mice.

#### Alveolar macrophages

There was minimal perturbation of alveolar macrophage (AM; Ly6G^-^ CD11b^-^ CD11c^+^ MHC II^hi^) numbers in the lungs of TIV-vaccinated and NC99-challenged mice (**Figure 1D**). In comparison, alum-vaccinated mice challenged with NC99 had 0.1X the number of AMs of the corresponding mock-challenged group. Our observed depletion of AMs in response to primary influenza infection only but not in immune-experienced hosts is in line with previous findings (75, 76, 81, 82). Of note, we observed a 0.6X reduction in AM numbers for OVA-sensitized mice compared to the PBS control group.

#### Eosinophil subsets

Next, we assessed if there were any phenotypic differences in the eosinophil compartment across vaccination and challenge regimens using CD101 and CD62L expression as described by Mesnil et al. to designate iEos (CD101^+^ CD62L^-^) and rEos (CD101^-^ CD62L^-^) subsets (62). Siglec-F expression levels could also be used to distinguish two discrete subsets, with total eosinophils bifurcating into a Siglec-F^hi^ subset and a Siglec-F^int^ subset. Previous literature has designated iEos as Siglec-F^hi^ and rEos as Siglec-F^int^, however we have observed that while Siglec-F^hi^ eosinophils are indeed predominantly iEos, a portion of CD62L^+^ rEos also exist within the population (**Figure S2A**) (62). Likewise, Siglec-F^int^ eosinophils were also composed of a mixture of iEos and rEos, with rEos outnumbering iEos (**Figure S2B**).

We used the ratio of iEos to rEos to quantify the phenotypic shifts in the lung eosinophil population. In our unchallenged PBS negative control, the iEos are outnumbered by the rEos and resulted in a ratio of 0.25 ± 0.04 (**Figures 1E–G**). In contrast, the OVA-sensitized positive control group had a large influx of iEos at 1 DPC following i.n. OVA challenge that shifted the iEos/rEos ratio upwards to 0.62 ± 0.14 (**Figure 1G**). Strikingly, the iEos/rEos ratio in our vaccinated and challenged groups, which had demonstrated increased infiltration of eosinophils as described above, was similar to that of the OVA-sensitized positive control while mock-challenged groups maintained ratios similar to the PBS negative control: NC99-challenged TIV (0.83 ± 0.26), TIV+Alum (0.54 ± 0.17), TIV+IMDQ (0.78 ± 0.51), and alum (0.36 ± 0.08); mock-challenged TIV (0.44 ± 0.05), TIV+Alum (0.50 ± 0.11), TIV+IMDQ (0.36 ± 0.09), and alum (0.25 ± 0.06) (**Figure 1G**). The phenotypic shifts in the composition of the bulk eosinophil population towards more iEos over rEos was consistently observed in all vaccinated and challenged groups, with no overt contribution of adjuvant (**Figures 1E, F**).

Eosinophils have also been classified into subsets on the basis of surface Siglec-F expression, with Siglec-F^hi^ eosinophils corresponding to more inflammatory functions and infiltration, while Siglec-F^int^ appears to be the homeostatic baseline state of eosinophils (62, 72). These observations appear to be consistent in both mouse models and human samples. We observed very few Siglec-F^hi^ eosinophils in the lungs in the absence of an inflammatory intranasal challenge, as demonstrated by our mock-challenged groups hovering around similar Siglec-F^hi^/Siglec-F^int^ ratios as our PBS control group (0.02 ± 0.01) (**Figures 1H–J**). In contrast, there was a dramatic influx of Siglec-F^hi^ eosinophils in the lungs of our OVA-sensitized mice upon OVA challenge, shifting the ratio of Siglec-F^hi^/Siglec-F^int^ eosinophils to 1.28 ± 0.55. Using the ratio of Siglec-F^hi^ to Siglec-F^int^, we saw similar patterns as seen with the iEos/rEos ratio analyses: groups that received both TIV-vaccination and NC99-challenge, irrespective of adjuvant, had higher ratios of Siglec-F^hi^/Siglec-F^int^ eosinophils compared to mock-challenged control groups: NC99-challenged TIV (0.49 ± 0.34), TIV+Alum (0.17 ± 0.17), TIV+IMDQ (0.79 ± 0.70), and alum (0.10 ± 0.04); mock-challenged TIV (0.06 ± 0.03), TIV+Alum (0.09 ± 0.06), TIV+IMDQ (0.12 ± 0.05), and alum (0.03 ± 0.01) (**Figure 1J**).

We evaluated if the influx of eosinophils in the lungs of vaccinated and challenged mice was a sex-dependent phenomenon (**Figure S3A**). Consistent with findings in female mice, male mice exhibited minimal (<5%) weight loss in our sublethal vaccine-matched challenge model (**Figure S3B**). Male mice were vaccinated with TIV and then challenged with 0.2 LD_50_ of NC99 demonstrated a 2.9-fold enrichment in lung eosinophils compared to the mock-challenged TIV-vaccinated group while no enrichment in eosinophils was observed in PBS-vaccinated mice after challenge, consistent with findings in female mice (**Figure S3C**). A 3.2-fold enrichment was also observed in neutrophils for TIV-vaccinated NC99-challenged mice compared to mock-challenged mice, while an 8.5-fold enrichment of lung neutrophils was observed in PBS-vaccinated NC99-challenged mice compared to the corresponding mock-challenged group (**Figure S3D**). No depletion in AMs was observed for TIV-vaccinated mice after the influenza challenge, while the absolute numbers of AMs in the lungs of PBS-vaccinated NC99-challenged mice was 0.6-fold that of the PBS-vaccinated mock-challenged group (**Figure S3E**). Similar to findings in female mice, there was a significant enrichment in absolute numbers of both iEos and rEos eosinophil subsets only in the vaccinated and challenged mice, but not in mock-challenged mice or in both PBS-vaccinated groups (**Figures S3F, G**). The iEos/rEos ratio was significantly elevated in TIV-vaccinated NC99-challenged male mice compared to mock-challenged PBS-vaccinated mice (**Figure S3H**). Interestingly, the iEos/rEos ratio was also elevated in PBS-vaccinated NC99-challenged male mice, which we did not observe in female mice (**Figure S3H**). As observed in female mice, Siglec-F^hi^ eosinophils, Siglec-F^int^ eosinophils, and the Siglec-F^hi^/Siglec-F^int^ ratio were substantially elevated in only TIV-vaccinated NC99-challenged male mice (**Figures S3I–K**).

### Unsupervised clustering reveals lung eosinophils following vaccine-matched challenge are highly heterogeneous

To complement findings from our manual gating analyses, we then used unsupervised clustering of our flow cytometry data using *CATALYST* to provide an unbiased insight into the phenotypic dynamics of the lung across all treatment groups and cell types.

**Figure 2:**
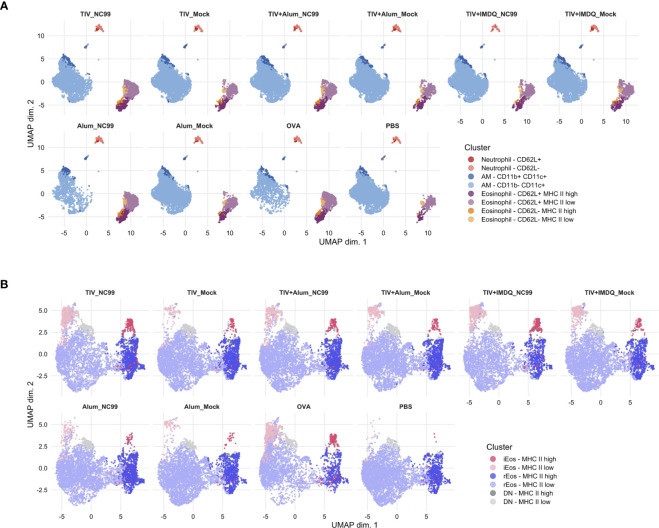
Unsupervised clustering reveals additional layers of heterogeneity in Siglec-F+ lung cells and eosinophils. **(A)** Live, singlet, Siglec-F^+^ cells or **(B)** total eosinophils (Ly6G^-^ CD11b^+^ CD11c^-^ CD125^int^ Siglec-F^+^) were clustered using the CATALYST package. Uniform manifold approximation and projection (UMAP) plots were generated using 10,000 cells.

Within Siglec-F^+^ cells, a large majority of the cells were AMs (72.34%), followed by eosinophils (25.17%) and neutrophils (2.49%) (**Figures 2A**, **S4**). A small subset (3.77% of Siglec-F^+^) of AMs also expressed CD11b in addition to CD11c, which might suggest infiltrating monocyte origin (83). Neutrophils were predominantly negative for CD62L expression, however a minor population expressed CD62L (0.32% of Siglec-F^+^). We noticed that there was heterogeneity within the eosinophil subset, clustering on the basis of MHC II expression levels and CD62L. A majority of eosinophils were CD62L^+^, likely corresponding to a more rEos-like phenotype while the CD62L^-^ eosinophils may correspond to iEos. Both CD62L^+^ and CD62L^-^ eosinophils were further bifurcated on the basis of MHC II expression, with low MHC II levels on a majority of cells in either subset. In line with previous manual gating results, a greater proportion of eosinophils were observed in TIV-vaccinated NC99-challenged mice compared to the corresponding mock-challenged control groups. AM depletion was also readily observed in unsupervised clustering results in the alum-only, NC99-challenged group and to a lesser extent in OVA-challenged mice.

We then used the unsupervised clustering analyses on the total eosinophil population (Ly6G^-^ CD11b^+^ CD11c^-^ CD125^int^ Siglec-F^+^) to dissect differences within our population of interest at a higher resolution (**Figures 2B**, **S5**). In the absence of other cell types, differential CD101 expression levels could be demarcated more clearly for binning of cells based on iEos and rEos phenotypic designations. Confirming results from manual gating analyses, a large majority of eosinophils were rEos with intermediate expression levels of Siglec-F (73.73%). Approximately a fifth (21.19%) of rEos had elevated expression of MHC II. iEos also expressed predominantly low levels of MHC II, although 28.55% of iEos were MHC II^hi^. 5.02% of eosinophils were double negative for both CD62L and CD101 expression. There was an enrichment in iEos in the TIV-vaccinated then NC99-challenged mice and OVA-sensitized mice, similar to observations for manual gating. Interestingly, the proportion of MHC II^hi^ versus MHC II^low^ within each eosinophil subset appeared consistent across treatment groups and inflammatory conditions.

### Mice with pulmonary eosinophilia mount effective antiviral responses and do not exhibit enhanced morbidity

Given the critical role of environmental cytokines in shaping cellular dynamics within tissues during inflammatory responses, we next characterized 26 chemokines and cytokines using a bead-based assay from lung homogenate supernatants collected at 7 DPC for the vaccinated and challenged mice and 1 DPC for the OVA-sensitized and PBS controls.

**Figure 3:**
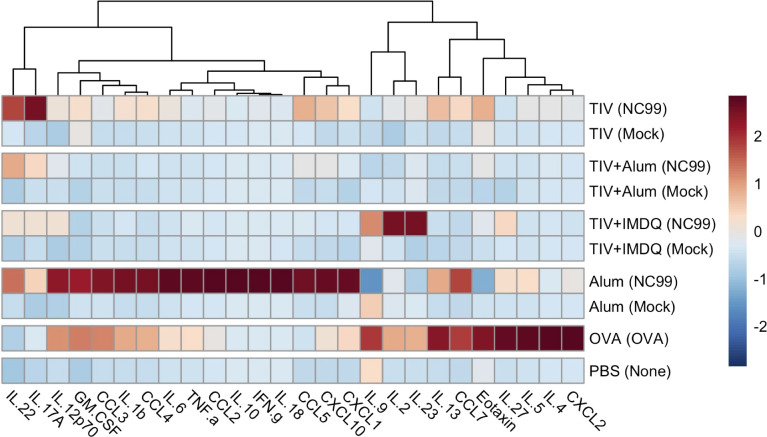
Distinct cytokine/chemokine profiles in lung homogenates are observed after vaccination and challenge. Left lung lobes were collected at 7 days post-challenge (DPC) for TIV, TIV+Alum, TIV+IMDQ, and Alum-vaccinated mice and at 1 DPC for OVA-sensitized and PBS control mice, then homogenized and clarified by centrifugation. Heatmap of net mean fluorescence intensity (MFI) for 26 cytokines/chemokines, *z*-scored by column.

From our analyses, no strong Type 1, 2, or 3 inflammatory signal was observed at 7 DPC in the lungs of mice that received both TIV-vaccination and NC99 challenge (**Figure 3**). In contrast, alum-only mice had potent induction of acute inflammation-associated cytokines (IL-1β, IL-6, and IL-18) alongside a clear and distinct Th1 module (IFN-γ, TNF-α). This is in line with expectations given that this is a primary infection for the alum-only mice since they were not primed with any influenza antigens. OVA-sensitized mice exhibited both a pro-inflammatory module (IL-1β, IL-6, TNF-α), albeit to a lesser extent compared to the alum group, alongside a Th2 cytokine module (IL-4, IL-5, IL-13, IL-9, IL-27, CCL11) at 1 DPC. IL-22 concentrations were modestly elevated in all groups receiving intranasal NC99 challenge (**Figure S6**). Interestingly, GM-CSF concentrations were highest in the alum-only NC99-challenged group as well as in the OVA-sensitized mice, but were undetectable in the majority of the vaccinated mice barring one mouse in the TIV-vaccinated group despite observing an influx of eosinophils into the lung (**Figure S6**). Concentrations of the chemokines CCL2, CCL3, CCL4, CCL5, CCL7, CXCL1, and CXCL10 were significantly higher in the alum-only NC99-challenged mice compared to all other groups (**Figure S6**). OVA-sensitized mice exhibited higher concentrations of CCL2, CCL3, CCL7, and CXCL2 (**Figure S6**). CCL5 and CCL7 concentrations in the lungs of TIV-vaccinated and challenged mice were both significantly elevated above mock-challenged counterparts, other TIV-vaccinated groups, and the PBS control group but below that of the alum-only NC99-challenged group (**Figure S6**). Of note, only the alum-only NC99-challenged group had significantly elevated concentrations of IL-10 out of all groups in the study (**Figures 3**, **S6**).

**Figure 4:**
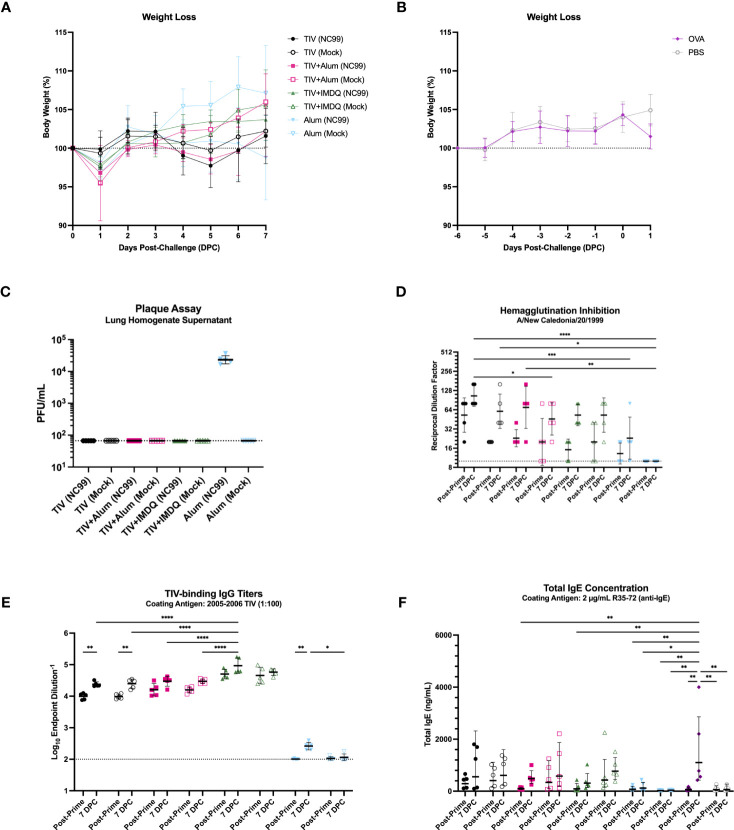
TIV-vaccinated mice are protected against severe morbidity and viral replication with intact serological responses. **(A)** Weight loss of TIV, TIV+Alum, TIV+IMDQ, and Alum-vaccinated mice through 7 DPC following intranasal, sublethal (0.2 LD_50_), vaccine-matched influenza challenge or allantoic fluid mock-challenge. **(B)** Weight loss of OVA-sensitized and PBS control mice through 1 DPC following intranasal OVA challenge or no challenge, respectively. Line represents the average of 5 mice. **(C)** Lung viral titers from clarified lung left lobe homogenate supernatants. Line denotes limit of detection (66.67 PFU/mL). **(D)** Serum hemagglutination inhibition titers against NC99. Line denotes limit of detection (titer of 10). **(E)** Serum TIV-binding IgG titers. Line demotes limit of detection (titer of 100). **(F)** Total IgE concentration in serum. Bars denote the **(C, D, F)** geometric mean ± geometric standard deviation or **(E)** mean ± standard deviation. Statistical significance in **(D–F)** was determined via two-way ANOVA with Tukey’s multiple comparisons test: ****P < 0.0001, ***P = 0.0001 to 0.001, **P = 0.001 to 0.01, *P = 0.01 to 0.05.

We also monitored weight loss as a measure of morbidity and mortality up to 7 days post-challenge. No mortality was observed, in line with the sublethal nature of the influenza challenge. Additionally, NC99-challenged mice lost minimal body weight irrespective of vaccination status, indicating little morbidity in this sublethal challenge model (**Figure 4A**). Minimal morbidity (<5% body weight loss) was also observed at 1 DPC for the control mice following OVA sensitization compared to un-challenged PBS controls (**Figure 4B**). Furthermore, all groups that received TIV vaccination did not have detectable lung virus titers, barring the alum-only group, indicating that all vaccinated animals were able to control viral replication by 7 days post-challenge (**Figure 4C**).

Using hemagglutination inhibition (HAI) titers as a readout of serum neutralization capacity for NC99, we demonstrated that all TIV-vaccinated mice generated modest HAI titers, while alum-only mice had predominantly undetectable HAI titers consistent with their antigen exposure history (**Figure 4D**). The low titers are in line with the suboptimal dose of vaccine administered, which is enough to induce seroconversion and vaccine-specific antibodies, but not enough to confer completely sterilizing immunity in the lungs (75, 76). Although HAI activity was low to modest for TIV-vaccinated mice, all mice that were vaccinated mounted robust vaccine-specific IgG responses (**Figure 4E**). Here we observe a clear adjuvant effect, where mice vaccinated with TIV+IMDQ exhibit significantly higher endpoint titers of TIV-binding IgG compared to mice that received TIV+Alum or TIV alone. The inclusion of alum as an adjuvant did not appear to elicit significantly higher IgG titers compared to TIV alone. No detectable TIV-specific IgG titers were measured in the alum-only groups, as expected. Given the eosinophil influx in the lungs in the absence of a strong Th2 cytokine module, we wanted to investigate if an allergy-like response was elicited in the serum by measuring total IgE (**Figure 4F**). All vaccinated mice generated a baseline level of total IgE by 2 weeks post-vaccination, which increased in concentration by 7 DPC. There appears to be no adjuvant effect on total IgE induction after TIV-vaccination. In line with previous literature, OVA-sensitized mice generated the highest titers of IgE compared to all other groups.

Furthermore, we used serum titers of IgG1 and IgG2a to indirectly discern host Th2 and Th1 skewing, respectively (**Figure S7**) (84–86). We observed robust induction of vaccine-specific IgG1 and IgG2a, in line with findings from total IgG analysis. TIV+Alum vaccination elicited the highest titers of IgG1, both at 2 weeks post-prime and 7 DPC, while TIV+IMDQ vaccination elicited the highest titers of IgG2a at both time points (**Figures S7A, B**). Unadjuvanted TIV-vaccinated mice had comparable IgG1 titers to the TIV+IMDQ vaccinated mice, and IgG2a titers that were significantly elevated above that of the TIV+Alum-vaccinated mice but substantially lower than that of the TIV+IMDQ-vaccinated mice. Using the IgG2a/IgG1 ratio as a metric to assess Th1/Th2 skewing, we observed that mice vaccinated with TIV+IMDQ had the highest ratio, TIV-only mice had the second highest ratio, followed by TIV+Alum mice (**Figure S7C**). This suggests that TIV vaccination in the absence of any adjuvant elicits a balanced Th1/Th2 response, while TIV+IMDQ skews towards Th1 and TIV+Alum skews towards Th2 (78, 79).

**Figure 5:**
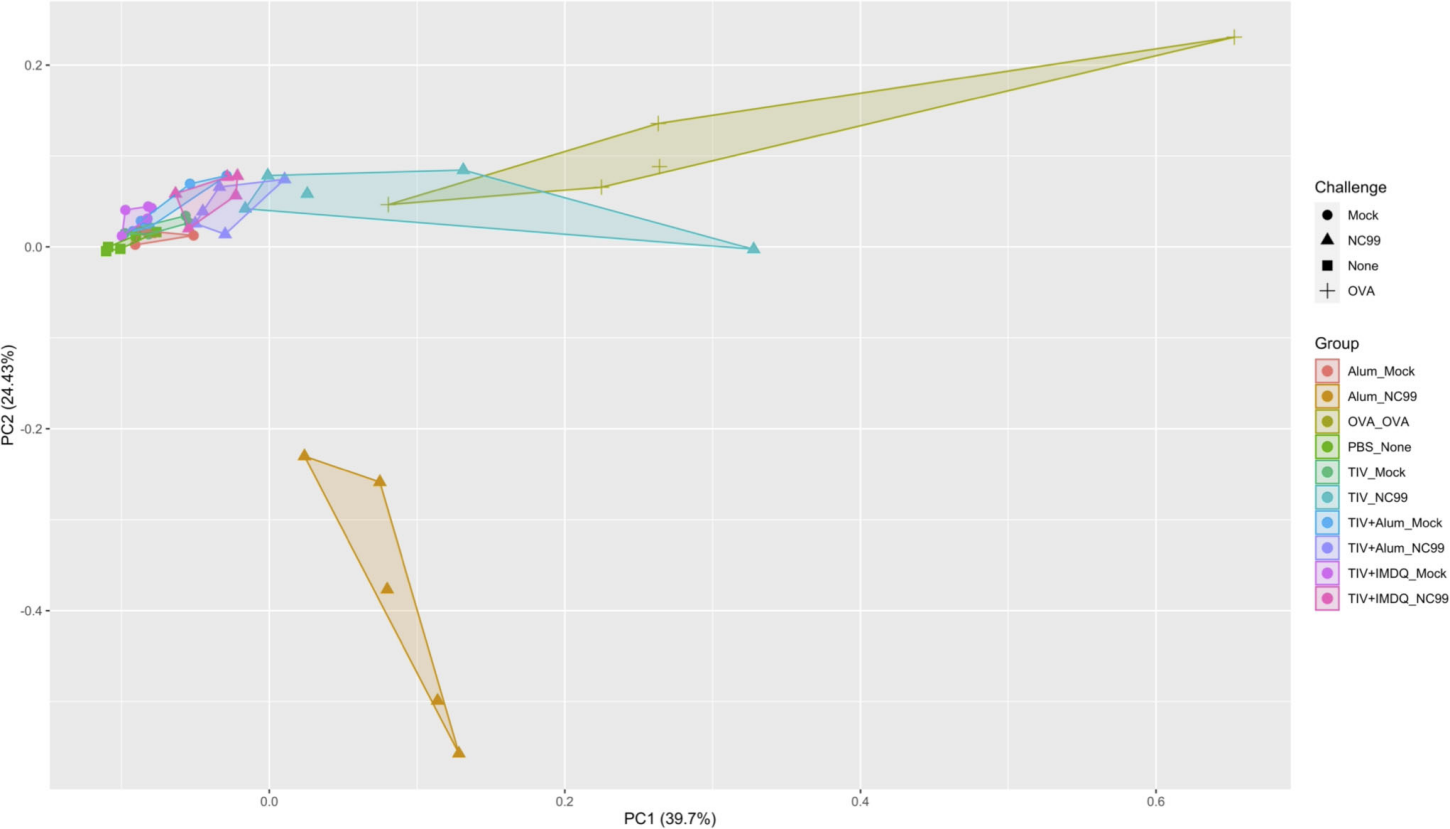
Principal component analysis reveals distinct immune profiles. Integration of flow cytometry populations of interest, lung viral titers, post-prime and post-challenge serology (TIV-binding IgG, HAI, total IgE), multiplex chemokine/cytokine analysis, and body weight via principal component analysis (PCA).

We then integrated individual-level data from all mice using principal component analysis (PCA) of 58 total parameters from flow cytometry, body weights, lung viral titers, TIV-specific IgG titers, HAI titers, total IgE concentrations, and cytokine/chemokine concentrations from lung homogenate supernatants (**Figure 5**). Distinct vaccination and challenge response profiles were observed after dimensionality reduction. Alum-vaccinated, NC99-challenged mice cluster further and separately from TIV-vaccinated, OVA-sensitized, and PBS control mice, driven by factors such as lung viral titers, IL-10, IL-18, IFN-γ, and loss of AMs (**Figure S8**). OVA-sensitized mice also clustered together away from the TIV-vaccinated mice, on the basis of Th2 cytokine concentrations and eosinophil absolute numbers. TIV-vaccinated, NC99-challenged mice clustered closer to the OVA-sensitized mice. TIV+Alum- and TIV+IMDQ-vaccinated mice clustered close to all mock-challenged groups and the PBS negative control.

### Intramuscular vaccination, but not prior infection, is required for pulmonary eosinophilia even after challenge with vaccine-mismatched influenza virus

**Figure 6:**
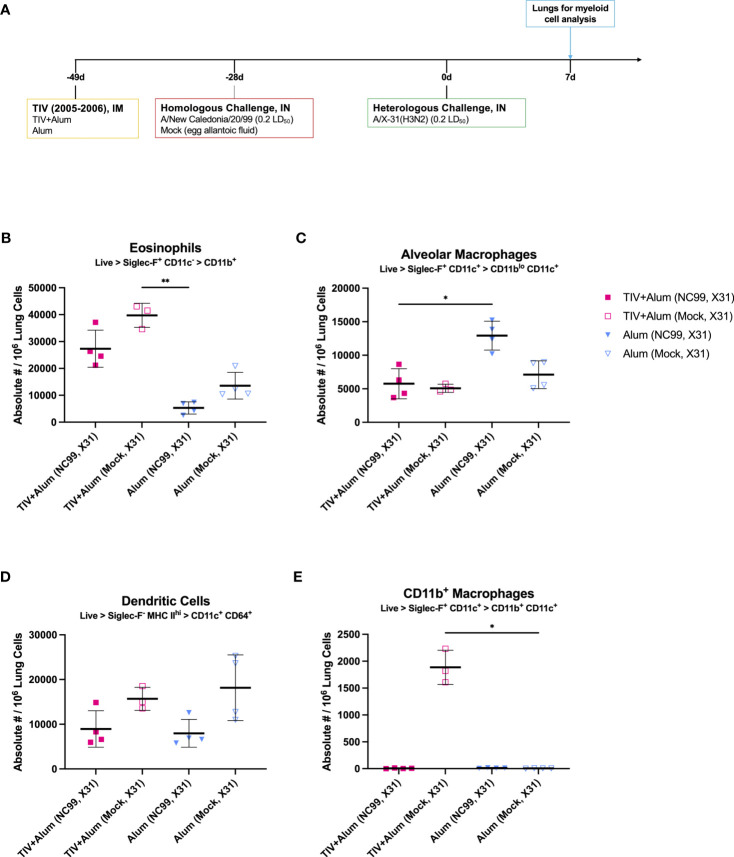
Vaccine-mismatched challenge results in differential lung myeloid cell dynamics depending on prior antigenic exposure. **(A)** Outline of study assessing impact of vaccine-mismatched challenge following TIV+Alum vaccination with or without vaccine-matched NC99-challenge on lung myeloid populations. Absolute numbers of **(B)** eosinophils, **(C)** alveolar macrophages, **(D)** dendritic cells, and **(E)** CD11b^+^ macrophages. Bars represent mean ± standard deviation in **(B–E)**. Statistical significance in **(B–E)** was determined via Kruskal-Wallis one-way ANOVA with Dunn’s multiple comparisons test: **P = 0.001 to 0.01, *P = 0.01 to 0.05.

We next expanded on the previously described breakthrough infection model by testing if pulmonary eosinophilia was also induced upon virus challenge with a vaccine-mismatched H3N2 influenza virus (**Figure 6A**). As an alternative to intramuscular TIV as the antigenic priming, we also tested if prior challenge with NC99, similar to TIV vaccination, could result in pulmonary eosinophilia upon rechallenge with a heterosubtypic H3N2 virus. After priming mice with TIV+Alum or alum alone, mice were sublethally (0.2 LD_50_) challenged with either the vaccine-matched NC99 or mock-challenged with egg allantoic fluid at 3 weeks post-vaccination. 4 weeks following homologous challenge, mice were then challenged with a sublethal dosage (0.2 LD_50_) of H3N2 A/X-31 (X31), which is a laboratory-adapted reassortant virus containing the HA and NA gene segments from H3N2 A/Hong Kong/1/1968 in the backbone of H1N1 (A/Puerto Rico/8/1934) and is mismatched from the TIV we use in our studies (87). Contrary to more recent H3N2 influenza viruses, this virus is able to efficiently infect mice and thereby cause severe morbidity for the chosen virus dose. At 7 DPC following heterosubtypic, vaccine-mismatched X31 challenge, we used flow cytometry to discern myeloid populations of interest (**Figure S9**).

Absolute numbers of eosinophils were significantly elevated in mice that were vaccinated and challenged, irrespective of challenge virus (**Figure 6B**). This elevation was not observed in alum-only mice even after a second intranasal influenza challenge with X31 after priming with NC99, suggesting that the nature of the primary antigenic exposure (intramuscular priming versus intranasal challenge) may be linked to distal influx of eosinophils in the lungs upon subsequent challenge. AM absolute numbers were highest in alum-vaccinated, NC99- and X31-challenged mice, at 1.8- to 2.6-fold higher than other experimental groups. It is likely that protection from vaccine alone or vaccine and NC99 challenge did not confer a sufficient amount of protection against loss of AMs (**Figure 6C**). Dendritic cells (DCs) were defined as Siglec-F^-^ MHC II^hi^ CD11c^+^ CD64^+^ cells, likely of monocyte origin (88). DCs were elevated in animals that were mock-challenged then X31-challenged, irrespective of initial vaccination regimen (**Figure 6D**). An influx of CD11b^+^ macrophages was only observed in TIV+Alum-vaccinated mice challenged with X31 7 weeks after initial vaccination and 4 weeks after mock intranasal challenge with egg allantoic fluid, and no other group (**Figure 6E**). These cells were Siglec-F^+^ CD11c^+^ similar to canonical AMs, but also exhibited surface expression of CD11b potentially indicating monocytic origin.

## Discussion

Dogmatically, eosinophils are associated with exacerbated disease after respiratory infection, however we did not observe a pathological contribution of eosinophils when recruited to the lungs during breakthrough infection of vaccinated mice. Our studies revealed a 2- to 4-fold enrichment in lung eosinophils of TIV-vaccinated NC99-challenged mice but not in egg allantoic fluid mock-challenged mice, irrespective of adjuvant. An enrichment in eosinophils was not observed in mice vaccinated with alum alone, suggesting the observed phenomenon is linked to vaccination or peripheral antigen exposure. Given that the numbers of lung eosinophils in vaccinated, egg allantoic fluid mock-challenged mice were similar to that of the un-challenged PBS-vaccinated control mice and the alum-only control mice, we are confident that the lung enrichment of eosinophils is not elicited by egg proteins in the vaccine or challenge virus. When focusing on eosinophil subsets, iEos and rEos have been well-characterized in the context of asthma as either exacerbators or controllers of lung inflammation, respectively, however their role has not been described in the context of breakthrough infection yet (37, 62, 72, 73). Moreover, it is still unclear if what we and others call rEos are *de facto* tissue-resident eosinophils or are a subset of eosinophils that is continuously being recruited into the tissue. For our report, we adhered to the currently described phenotypes for iEos (CD101^+^ CD62L^-^) and rEos (CD101^-^ CD62L^+^) in the literature but uncovered further heterogeneity in the eosinophil compartment following vaccine-matched challenge through unsupervised clustering. We observed a CD101^+^ CD62L^+^ double-positive eosinophil population containing a small CD101^hi^ population within, both of which have not been described in the literature and will be the subject of phenotypic interrogation in future studies.

We noticed the composition of the eosinophil compartment shifted after infection: mice that received both vaccine and challenge had an influx of iEos, a subset typically vastly outnumbered by rEos in the lung, resulting in an iEos/rEos ratio similar to that of the OVA allergic-sensitized mice but without evidence of enhanced morbidity, aberrant cytokine signatures, or uncontrolled viral replication at 7 DPC, irrespective of adjuvant. Previous studies by our group also demonstrated that lung eosinophil influx is vaccine dose-dependent and correlated with protection rather than immune pathology, in stark contrast to what has been reported for FI-RSV (75, 76). In the study detailed herein, all TIV-vaccinated mice were able to generate vaccine-specific IgG responses and had modest HAI titers, in accordance with our breakthrough infection model. A low level of serum IgE was detected after vaccination with TIV or OVA but not after alum alone, although IgE concentrations were the highest for OVA-sensitized mice. We observed in this study that a baseline, low level of IgE is generated in response to peripheral antigen exposure irrespective of antigen and adjuvant. When assassin lung cytokine modules, no overt Th2 cytokine module was observed in vaccinated and challenged mice exhibiting eosinophilia, a marked difference from small rodent models of VAERD which are characterized by high levels of IL-4, IL-5, and IL-13 and weight loss (54). Collectively, this indicates that the lung eosinophil influx we observe in vaccinated animals likely is not pathogenic like in the case of VAERD but correlates instead with viral clearance, systemic protection, and functional serological response. The specific contribution of each individual eosinophil subset to protection or pathology will be the focus of future investigations.

We interrogated lung homogenate supernatants to understand the potential contribution of lung cytokines and chemokines to local inflammation and observed cellular effects. We predominantly observed cytokines associated with acute inflammation (IL-1β, IL-6, and/or IL-18) for the alum-vaccinated NC99-challenged mice at 7 DPC and the OVA-sensitized mice at 1 DPC, and cytokines for Type 1 (IFN-γ, TNF-α) and Type 2 (IL-4, IL-5, IL-13, IL-9, CCL11) responses respectively (89–92). This is in line with expectations given the alum-vaccinated mice have no pre-existing antigen-specific immunity, so the NC99 challenge represents a primary viral infection, canonically a Type 1-skewed, whereas OVA-sensitization is well documented to induce a Type 2 response (89, 93). Lung IL-27 concentrations were significantly higher in OVA-sensitized mice at 1 DPC, potentially as a feedback mechanism to limit Type 2 immunopathology in the lung (94, 95). Alum-vaccinated NC99-challenged mice and OVA-sensitized mice also both had elevated concentrations of GM-CSF, CCL2, CCL3, and CCL7 at the respective timepoints. GM-CSF promotes the influx of myeloid cells, CCL2 is known to recruit monocytes, CCL3 has a role in lung inflammation and lymphocyte recruitment, and CCL7 can be linked to acute neutrophilic lung inflammation (96–100). Collectively, these chemokine signatures are in line with the nature of the acute inflammation we observed in the lung for these two groups.

Interestingly, we saw elevated concentrations of IL-2, IL-9, and IL-23 in TIV+IMDQ mice that were challenged with NC99 at 7 DPC but not in TIV+IMDQ mice that were mock-challenged, although not statistically significant (**Figures 3**, **S6**). Given that a balanced production of IL-2 and IL-23 from CD103^+^ lung DCs can mediate CD4^+^ T cell differentiation in *Aspergillus*-infected mice, we may investigate our observed cytokine signature in future studies while considering the contribution of DCs and T cells (101).

CCL5 and CCL7 concentrations were significantly higher in TIV-vaccinated NC99-challenged mice, which had the highest fold-increase in lung eosinophils of the TIV-vaccinated mice, compared to mock-challenged mice. IL-22, CCL11, and CXCL1 concentrations were elevated in TIV-vaccinated NC99-challenged mice as well, but not at statistically significantly higher levels above the corresponding mock-challenged mice (**Figures 3**, **S6**). Both CCL7 and CXCL1 can act as chemoattractants for neutrophils, corroborating the 2.6-fold increase in the TIV-vaccinated NC99-challenged mice compared to their mock-challenged counterparts (**Figure 1C**) (100, 102). However, TIV-vaccinated mice have similar numbers of lung neutrophils as TIV+Alum and TIV+IMDQ vaccinated mice after the sublethal NC99 challenge, suggesting an additional role for lung CCL7 in this context beyond neutrophil recruitment (**Figure 1C**). CCL5, also known as RANTES (Regulated upon Activation, Normal T Cell Expressed and Presumably Secreted), potently attracts monocytes, eosinophils, and T cells (103). Since no major CCL11 or IL-5 signature was observed in TIV-vaccinated NC99-challenged mice, we may be observing CCL5-mediated recruitment of eosinophils instead of canonical Type 2 cytokine-driven eosinophilia. Although the lung eosinophil influx we observe here does not appear to correspond to VAERD or increased morbidity, higher lung levels of CCL5 have positively correlated with disease severity in the lungs of children infected with RSV (103). However, our simultaneous observation of increased lung IL-22 concentrations may be ameliorating immunopathology, as previous research has demonstrated IL-22-mediated reduction of lung injury and promotion of airway repair in the context of sublethal viral infection (104, 105). We are currently conducting additional studies to dissect if the observed lung eosinophil influx is pathogenic or not at greater depth and dimension.

Given the nature of respiratory viral infections, vaccine design has been largely focused on eliciting a robust and neutralizing Th1-centric response, with little to no Th2 responses elicited (90, 106). A growing body of literature suggests that a more balanced Th1/Th2 response may be more beneficial for not only the acute response to vaccination, but also for long-term durability and rapid tissue repair in the event the vaccine-matched virus is encountered and is able to induce a breakthrough infection (107–111). Type 2 immune-biased hosts, such as asthmatics, in some instances have better outcomes in response to primary viral infections such as SARS-CoV-2 or influenza, perhaps due to IL-13 remodeling of the lung (112–115). Higher counts of circulating eosinophils were demonstrated to correlate with reduced mortality and shorter hospital stays in a cohort of hospitalized COVID-19 patients (116). However, the opposite has also been observed in clinical cohorts where IL-4/13 receptor blockade can reduce disease severity in SARS-CoV-2 infection (117). Despite the induction of eosinophils, typically associated with Type 2 responses, we did not see a clear role for IL-4, IL-5, or IL-13 in our data at the given time points. We also reported that the treatment groups with the most lung eosinophils did not have a strongly IgG1-skewed serum antibody response to the vaccine, further decoupling the observed phenomenon from aberrant induction of Th2 immunity (**Figure S7**). We speculate the non-pathological enrichment of iEos, located in the alveolar space, during breakthrough infection may be for both direct and indirect viral control. Eosinophils stimulated *in vitro* with Type 1 stimuli such as IFN-γ or IFN-γ with E. coli exhibit upregulation of transcripts for co-stimulatory molecules such as *Cd80* and *Cd86*, CD8 T cell chemotaxis-inducing cytokines like *Cxcl9* and *Cxcl10*, and the immunomodulatory *Cd274* (PD-L1) (63). *In vivo*, eosinophils have been shown to have direct antiviral effects in the context of primary influenza infection and promote clearance, as well as CD8 T cell activation (118). iEos specifically have been demonstrated to upregulate *Il6*, *Il13ra1*, and other pro-inflammatory genes (62). We will investigate whether or not environmental signals during breakthrough viral infection in the lung lead to the recruitment of eosinophils to mediate viral clearance, activation of adaptive immune cells, or temper aberrant T cell-mediated immunopathology. Nevertheless, our current study and previous work highlight a possibly protective role for eosinophils in hosts with pre-existing vaccine-mediated immunity upon breakthrough infection, potentially as a novel cellular target in vaccination strategies against viruses that are able to escape from neutralizing antibodies.

Our investigation is the first to subset lung eosinophils in the context of vaccination with respiratory viral challenge, demonstrating a potentially protective role for eosinophils in local mucosal sites of infection after distal intramuscular priming, but not after primary or repeated infection in mice. This phenomenon was sex-independent, observed in both female and male mice. Given reports of iEos and rEos in human clinical samples, we anticipate that human eosinophils may also play a role in the antiviral response following a vaccine-matched infection (65, 74, 119).

A limitation of our study is the inclusion of blood-derived eosinophils and cytokines in the lung, as we did not perfuse samples prior to processing for downstream analyses. However, phenotyping studies by groups have demonstrated CD101 is not present in circulating eosinophils in the blood but may be acquired in the lung, suggesting the CD101^+^ eosinophils we observe in the lung are likely to have shifted to a more physiologically relevant phenotype in the specific tissue niche rather even if they are of blood origin, away from the baseline homeostatic state (68). Although no overt Th1 or Th2 cytokine module in TIV-vaccinated and NC99-challenged mice was detected at 7 DPC in lung homogenates and balanced IgG2a/IgG1 antibody induction was observed despite lung eosinophilia, we speculate that recruitment signals may occur earlier in the course of infection and are currently conducting longitudinal kinetic studies to evaluate this and to decide which mechanistic studies are needed to reveal key drivers of non-pathological eosinophilia in our breakthrough model. We did not perform direct T cell analyses for this suite of studies, however our group has previously demonstrated that during sublethal, vaccine-matched influenza infection, T cells are not robustly recruited or activated (75). This is in part due to the nature of inactivated influenza vaccines, which results in antibody-dominated immune control of viral infection rather than cell-mediated immunity. We are aware that our observations may be specific to the mouse vaccination/challenge model. Therefore we will also investigate if similar eosinophil recruitment events occur after vaccination in other species, including humans, or with other vaccine-virus pairs in future experiments. We also plan on incorporating the FI-RSV VAERD model or other animal models of VAERD as comparators to assess how the subsets of eosinophils differ in phenotype and function under non-pathological and pathological breakthrough infection.

A more mechanistic understanding of each eosinophil subset and how they contribute to outcomes following vaccine-matched respiratory viral infection is needed, alongside more descriptive phenotyping markers to understand why eosinophils are recruited to the lung in this context. Future directions include investigating if lung enrichment of eosinophils is antigen-agnostic training of lung innate immune cells by using different vaccines and respiratory viruses, examining the immune circuits contributing to eosinophil recruitment, and deep phenotyping of eosinophils after different types of inflammatory insults. Functional analyses of eosinophil subsets derived from different inflammatory conditions (e.g. breakthrough infection versus allergic sensitization) will also be conducted by subjecting them to cytokine secretion and degranulation assays. Follow-up mechanistic studies using eosinophil deficient or knock-out mice, or perturbations in pathways that result in abrogation of eosinophil activation and recruitment, may be necessary to fully understand the functional contributions of each subset. We hope to expand our findings to human samples as well to assess the significance of eosinophil subsets in human immune responses to respiratory viral infection after antigen-matched vaccination, and whether or not they mediate protection or enhance pathology, especially in the context of breakthrough infections. It has become increasingly clear that eosinophils play multiple roles in both homeostasis and disease, and our preclinical animal model provides a tool to investigate these features for eosinophils in the context of vaccination and respiratory infection.

## Data availability statement

The raw data supporting the conclusions of this article will be made available by the authors, without undue reservation.

## Ethics statement

The animal study was reviewed and approved by IACUC of the Icahn School of Medicine at Mount Sinai.

## Author contributions

LAC, AC, and MS conceived and conceptualized the work and strategy. LAC, AC, RR, PW, MN, SJ, and YC performed experiments and analyzed and interpreted data. YC and BGD provided specific materials. LAC and MS wrote the manuscript. All authors critically reviewed the manuscript. All authors contributed to the article and approved the submitted version.
