# Supplementary material for: Influenza breakthrough infection in vaccinated mice is characterized by non-pathological lung eosinophilia
Source: Front Immunol. 2023 Aug 4;14:1217181. doi: 10.3389/fimmu.2023.1217181 (PMC10437116; doi:10.3389/fimmu.2023.1217181)
Supplement: Supplementary file 1 [file Image_1.pdf]

Supplementary Material

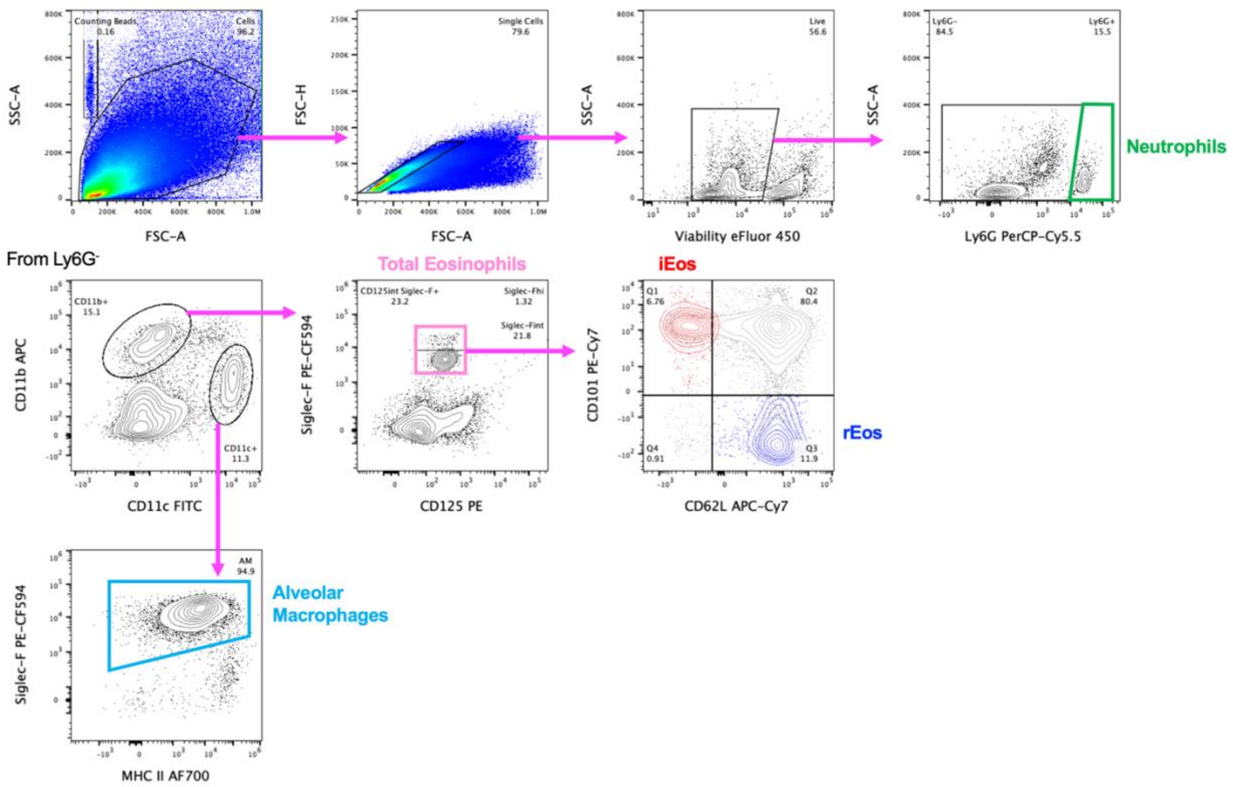

Figure S1. Gating strategy for eosinophil phenotyping study. Representative gating strategy using data from a TIV-vaccinated, NC99-challenged mouse.

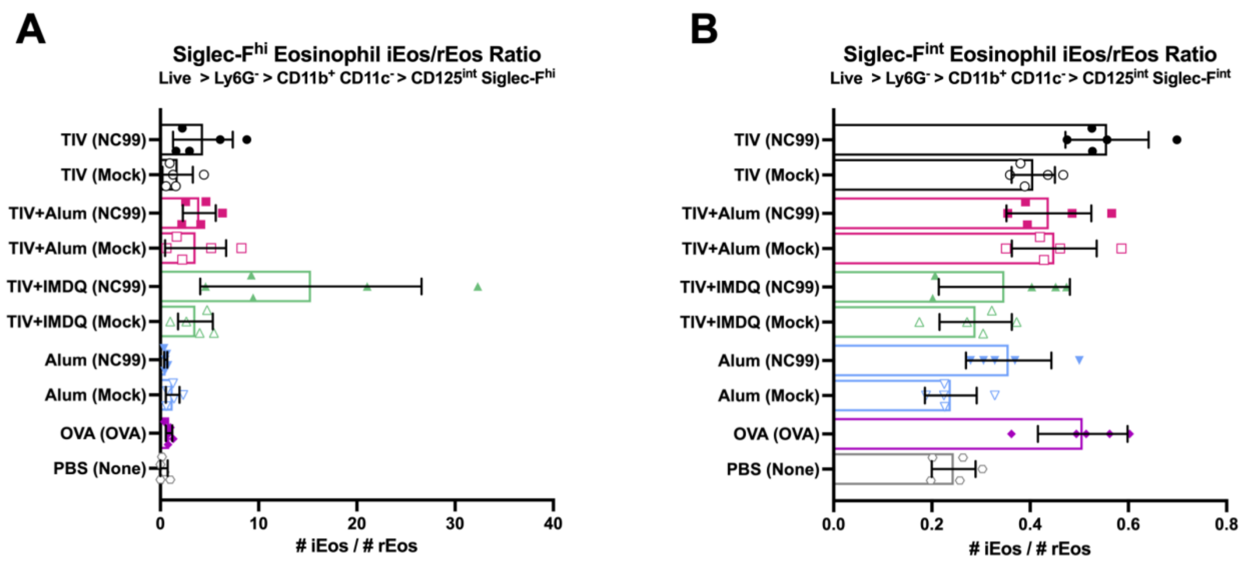

Figure S2. iEos/rEos ratio in Siglec-F^{hi} and Siglec-F^{int} eosinophil subpopulations.

The ratio of inflammatory eosinophils (iEos, CD101^{+} CD62L^{-}) to resident eosinophils (rEos, CD101^{-} CD62L^{+}) in (**A**) Siglec-F^{hi} and (**B**) Siglec-F^{int} eosinophil subpopulations. Ratios were calculated using the absolute numbers of each population in the right lung lobes. In (**A-B**), bars describe mean ± standard deviation.

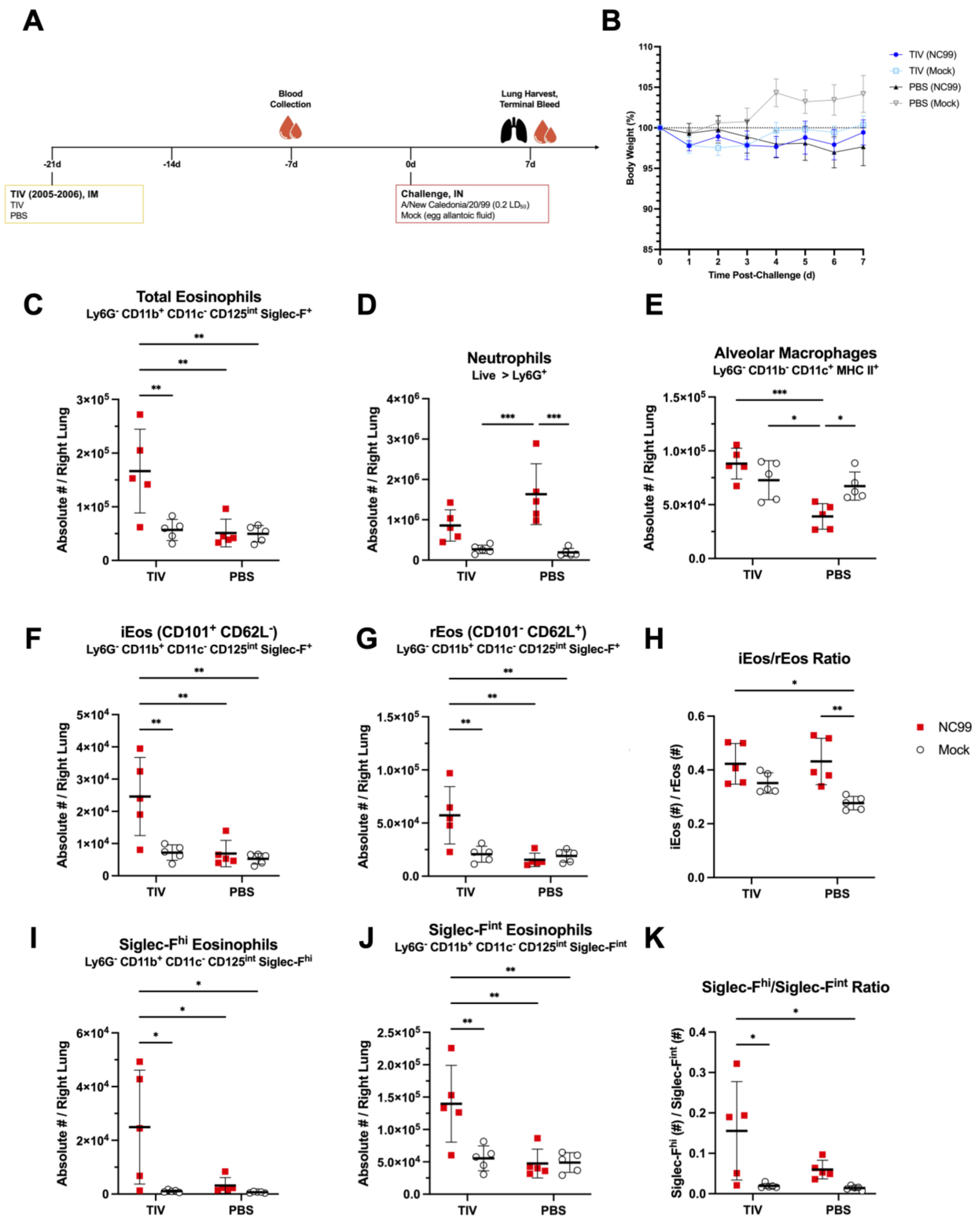

Figure S3. Lung eosinophil influx is observed in male BALB/cJ mice. (**A**) Outline of study assessing if lung eosinophilia is observed in TIV-vaccinated, NC99-challenged male mice. (**B**) Weight loss through 7 DPC following vaccine-matched, sublethal (0.2 LD_{50}), intranasal NC99 challenge. Absolute number of (**C**)

total eosinophils, (**D**) neutrophils, (**E**) alveolar macrophages, (**F**) iEos, and (**G**) rEos in right lung lobes. Ratio of (**H**) iEos/rEos. Absolute number of (**I**) Siglec-F^{hi} and (**J**) Siglec-F^{int} eosinophils in right lung lobes. Ratio of (**K**) Siglec-F^{hi}/Siglec-F^{int} eosinophils. Ratios were calculated using the absolute numbers of each population in the right lung lobes. For (**C-K**), bars indicate mean ± standard deviation. Statistical significance in (**C-K**) was determined via two-way ANOVA with Tukey's multiple comparisons test: ***P = 0.0001 to 0.001, **P = 0.001 to 0.01, *P = 0.01 to 0.05.

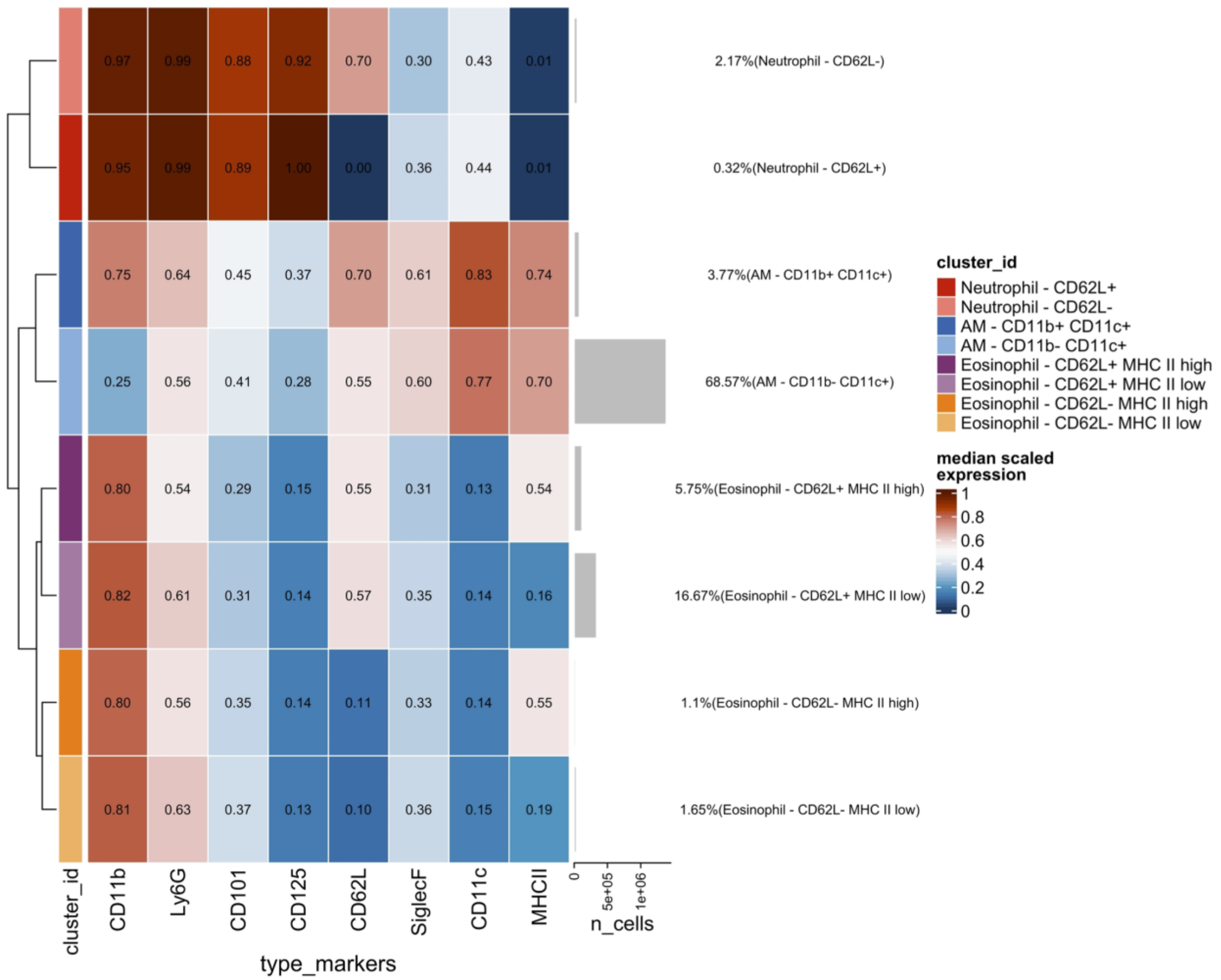

Figure S4. Cluster-level surface marker expression and proportion of Siglec-F$^+$ cells. 10,000 live, singlet, Siglec-F$^+$ cells were subjected to unsupervised clustering using the *CATALYST* package. Heatmap depicts relative surface marker expression is *z*-scored by column and bars denote proportion of total cells analyzed per cluster.

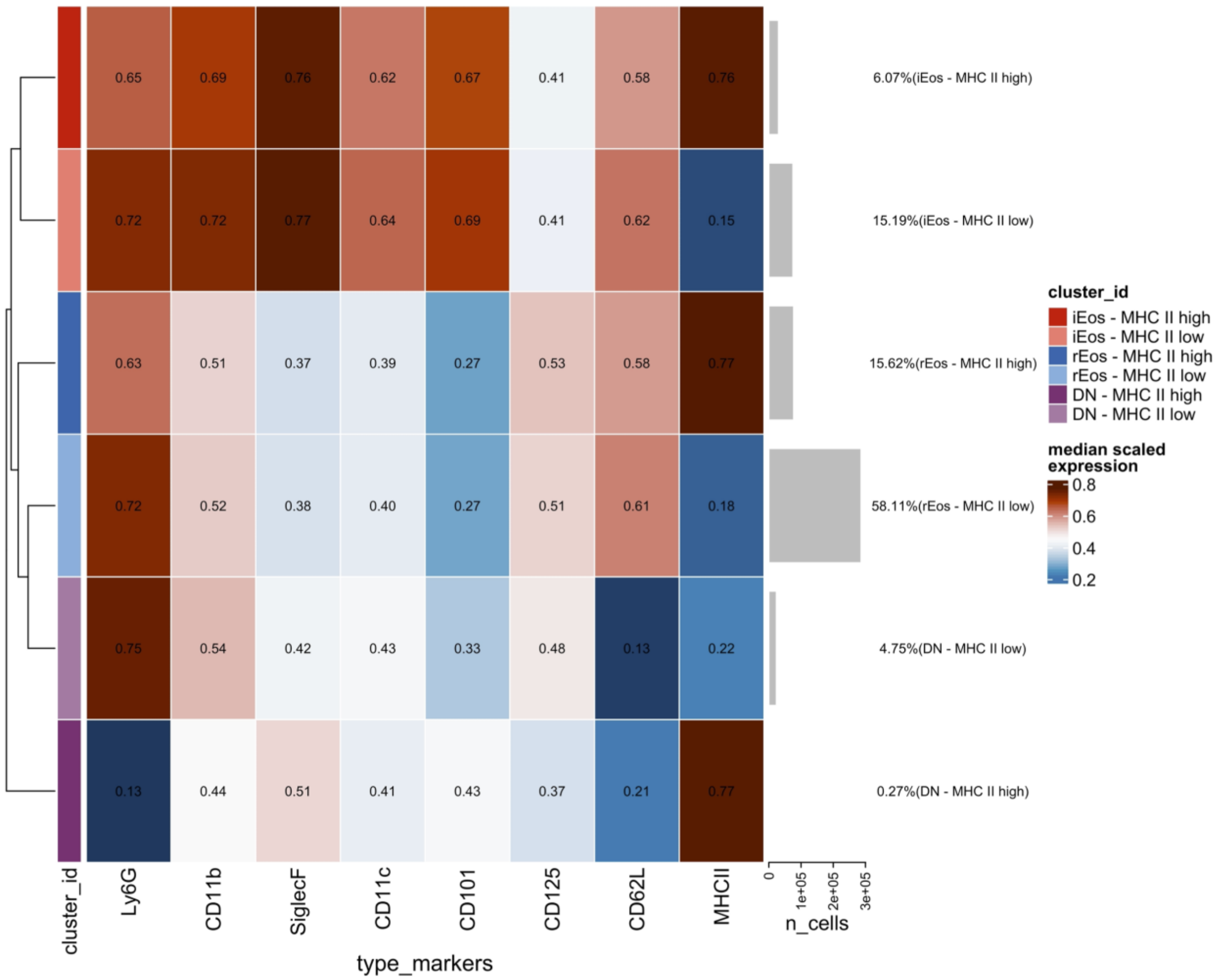

Figure S5. Cluster-level surface marker expression and proportion of total eosinophils. 10,000 live, singlet, Ly6G^{-}, CD125^{int} Siglec-F^{+} cells were subjected to unsupervised clustering using the *CATALYST* package. Heatmap depicts relative surface marker expression is *z*-scored by column and bars denote proportion of total cells analyzed per cluster.

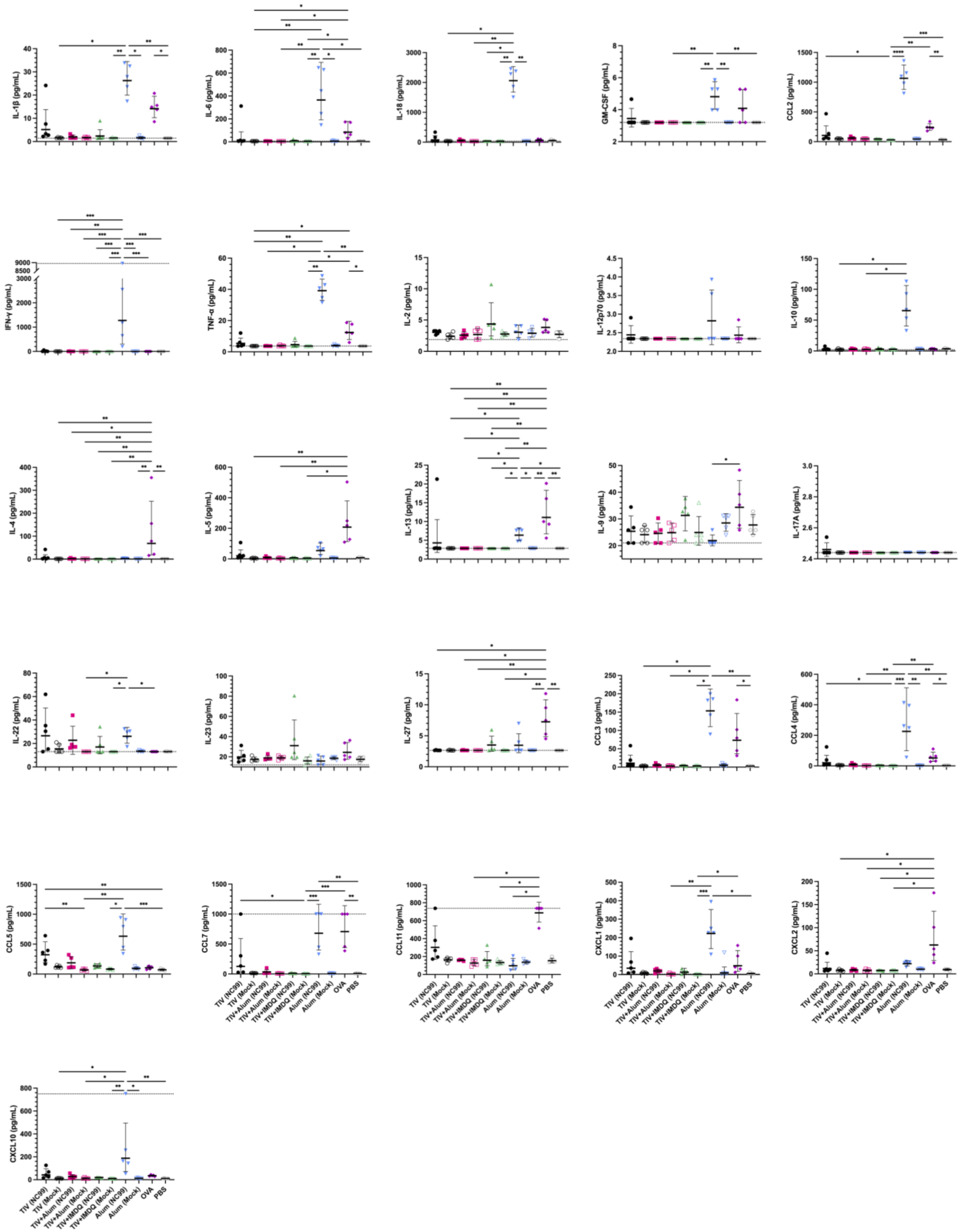

Figure S6. Concentrations of cytokines and chemokines in clarified lung homogenate supernatants. Concentrations (pg/mL) were extrapolated in the acquisition program from in-assay

standard curves generated using the standards provided by the manufacturer. Lines denote lower limit of quantitation (LLOQ) and upper limit of quantitation (ULOQ). Values above or below the limits of quantitation were arbitrarily set to the ULOQ and LLOQ, respectively. Bars denote the geometric mean ± geometric standard deviation. Statistical significance was determined via Kruskal-Wallis one-way ANOVA with Dunn's multiple comparisons test: ****$P < 0.0001$, ***$P = 0.0001$ to 0.001, **$P = 0.001$ to 0.01, *$P =$ 0.01 to 0.05.

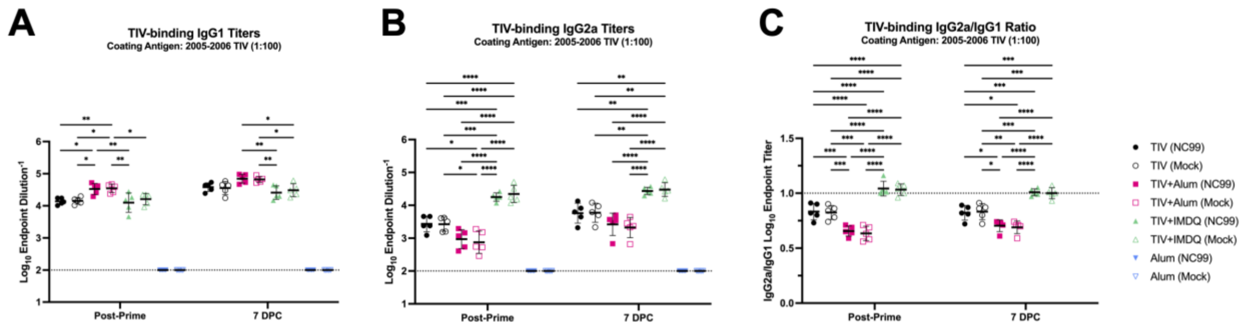

Figure S7. Induction of TIV-specific IgG1 and IgG2a in mice 2 weeks after priming and 7 DPC. (**A**) Serum TIV-binding IgG1 titers. (**B**) Serum TIV-binding IgG2a titers. (C) IgG2a/IgG1 ratio in serum. Ratio was calculated by dividing the IgG2a titer by the IgG1 ratio, matching time points. Line denotes (**A**, **B**) limit of detection (titer of 100) or (**C**) an IgG2a/IgG1 ratio of 1. Bars denote the mean ± standard deviation. Statistical significance was determined via two-way ANOVA with Tukey's multiple comparisons test: ****P < 0.0001, ***P = 0.0001 to 0.001, **P = 0.001 to 0.01, *P = 0.01 to 0.05. Alum-only mice were undetectable via both IgG1 and IgG2a assays and omitted from statistical analysis and IgG2a/IgG1 ratio calculation.

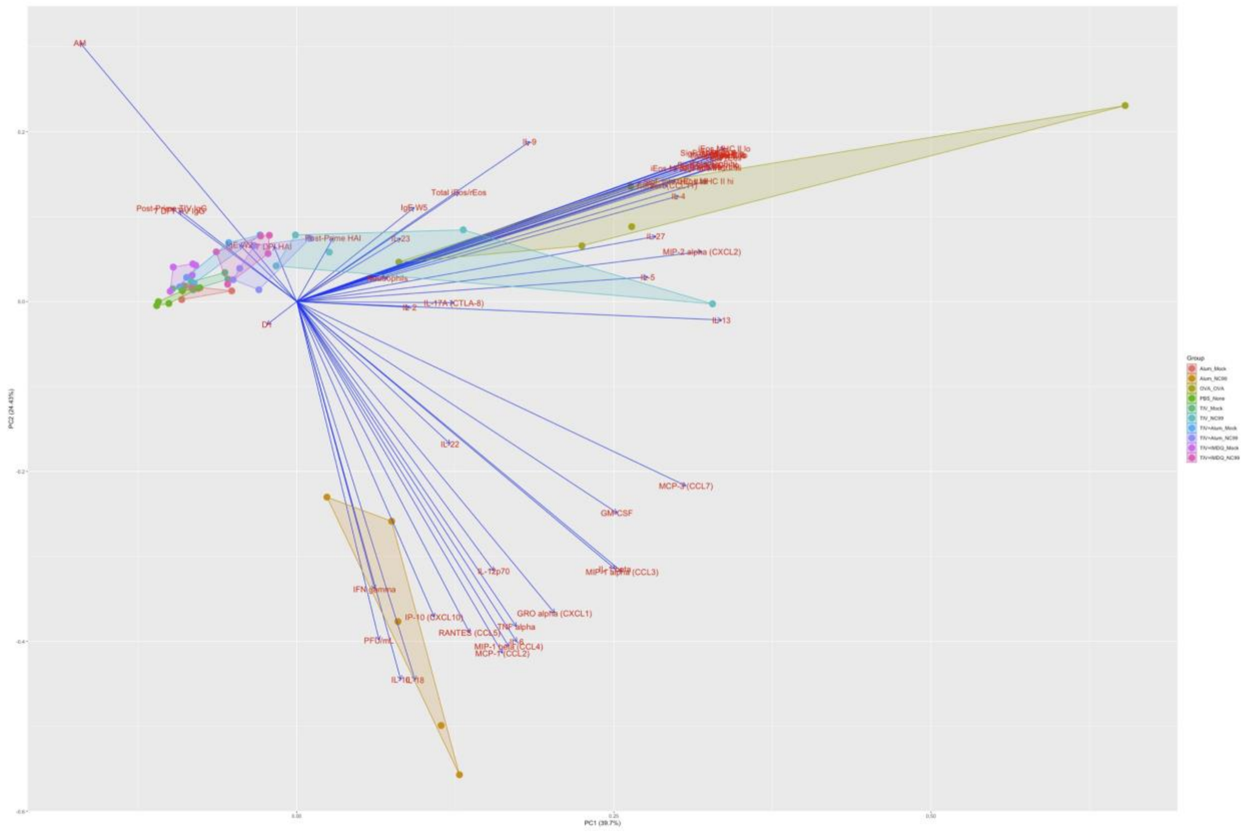

Figure S8. Loadings plot from principal component analysis. PCA plot generated using *prcomp* with loadings vectors overlaid.

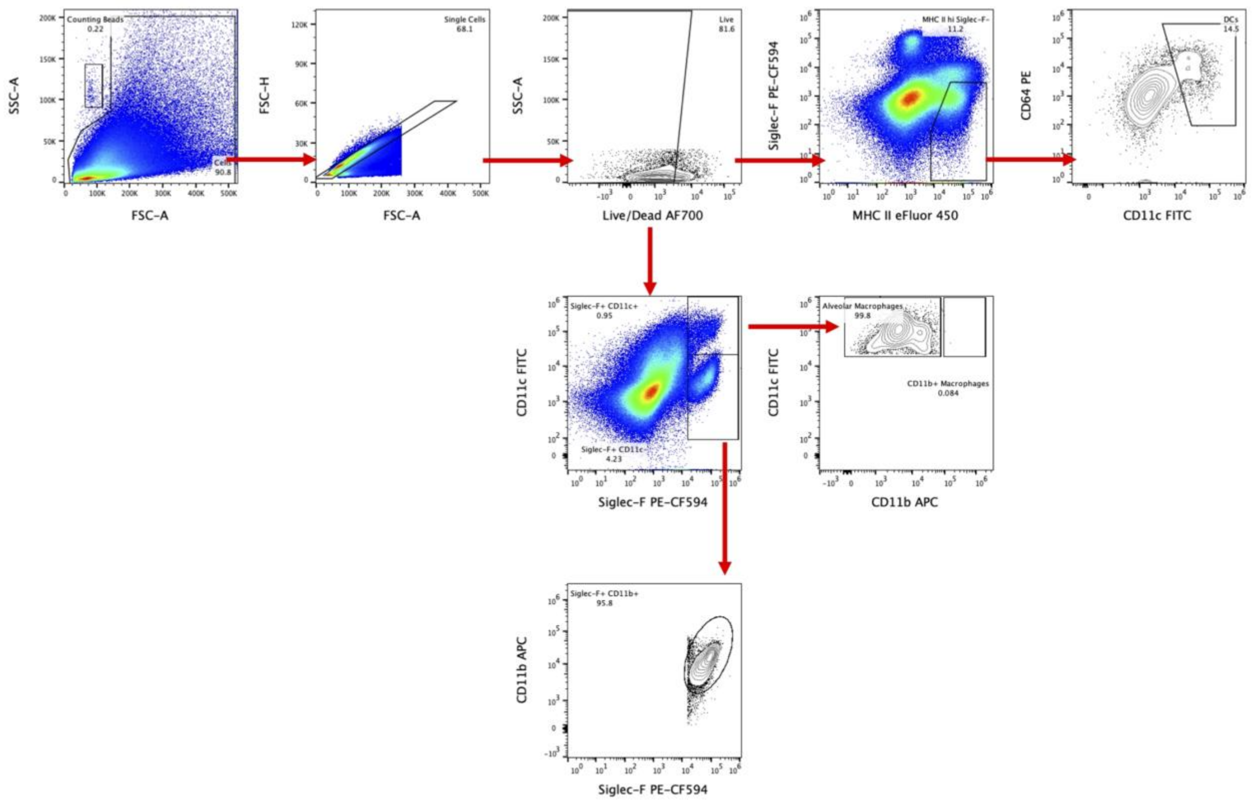

Figure S9. Gating strategy for vaccine-mismatched challenge study. Representative gating strategy from a TIV+Alum-vaccinated, NC99-experienced, X31-challenged mouse.
